# Changes in the Aggregation Behaviour of Zinc Oxide Nanoparticles Influenced by Perfluorooctanoic Acid, Salts, and Humic Acid in Simulated Waters

**DOI:** 10.3390/toxics12080602

**Published:** 2024-08-18

**Authors:** Anwar Ul Haq Khan, Yanju Liu, Ravi Naidu, Cheng Fang, Ho Kyong Shon, Huiming Zhang, Rajarathnam Dharmarajan

**Affiliations:** 1 Global Centre for Environmental Remediation (GCER), College of Engineering Science and Environment, The University of Newcastle, Callaghan, NSW 2308, Australia; anwar.khan@uon.edu.au (A.U.H.K.); ravi.naidu@newcastle.edu.au (R.N.); cheng.fang@newcastle.edu.au (C.F.); 2 crc for Contamination Assessment and Remediation of the Environment (crcCARE), ATC Building, The University of Newcastle, Callaghan, NSW 2308, Australia; 3 School of Civil and Environmental Engineering, University of Technology Sydney (UTS), City Campus, Broadway, Sydney, NSW 2007, Australia; hokyong.shon-1@uts.edu.au; 4 Electron Microscope and X-ray (EMX) Unit, The University of Newcastle, Callaghan, NSW 2308, Australia; hui-ming.zhang@newcastle.edu.au; 5 Australian Centre for Water and Environmental Biotechnology (ACWEB), The University of Queensland, Brisbane, QLD 4072, Australia; r.dharmarajan@uq.edu.au

**Keywords:** zinc oxide nanoparticles, perfluorooctanoic acid, humic acid, electrolytes, adsorption, zeta potential, aggregation

## Abstract

The increasing utilization of zinc oxide nanoparticles (ZnO-NPs) in many consumer products is of concern due to their eventual release into the natural environment and induction of potentially adverse impacts. The behaviour and environmental impacts of ZnO-NPs could be altered through their interactions with environmentally coexisting substances. This study investigated the changes in the behaviour of ZnO-NPs in the presence of coexisting organic pollutants (such as perfluorooctanoic acid [PFOA]), natural organic substances (i.e., humic acid [HA]), and electrolytes (i.e., NaCl and CaCl_2_) in simulated waters. The size, shape, purity, crystallinity, and surface charge of the ZnO-NPs in simulated water after different interaction intervals (such as 1 day, 1 week, 2 weeks, and 3 weeks) at a controlled pH of 7 were examined using various characterization techniques. The results indicated alterations in the size (such as 162.4 nm, 1 day interaction to >10 µm, 3 weeks interaction) and zeta potential (such as −47.2 mV, 1 day interaction to −0.2 mV, 3 weeks interaction) of the ZnO-NPs alone and when PFOA, electrolytes, and HA were present in the suspension. Different influences on the size and surface charge of the nanoparticles were observed for fixed concentrations (5 mM) of the different electrolytes. The presence of HA-dispersed ZnO-NPs affected the zeta potential. Such dispersal effects were also observed in the presence of both PFOA and salts due to their large aliphatic carbon content and complex structure. Cation bridging effects, hydrophobic interactions, hydrogen bonding, electrostatic interactions, and van der Waals forces could be potential interaction forces responsible for the adsorption of PFOA. The presence of organic pollutants (PFOA) and natural organic substances (HA) can transform the surface characteristics and fate of ZnO-NPs in natural and sea waters.

## 1. Introduction

Zinc oxide nanoparticles (ZnO-NPs) are among the most abundantly synthesized metal oxide-based nanoparticles, with an estimated annual global market of USD 3600 million and a global yield of 10 Mt [1]. This is due to their popular application in cosmetics, electronics, medical dressings, paints, textiles, UV filters, and other products [2,3,4]. Among the several causes of toxicity due to exposure to ZnO-NPs in water systems, the three major mechanisms for the toxic effects to the ecosystems are (1) photocatalytic activity and generation of reactive oxygen species, (2) release of dissolved zinc ions, and (3) attachment of the ZnO-NPs to the cell wall through electrostatic interactions, damaging the DNA structure and causing oxidative stress, etc. However, due to their small size, large surface-to-mass ratio, and strong ability to pose toxic effects (i.e., an impact on microbial and aquatic communities, such as DNA damage, oxidative stress, and soil and plant transfer) in the environment, ZnO-NPs can pose a problem for ecological receptors in water, soil, and human health [5,6,7,8]. The occurrence of ZnO-NPs in environmental samples and wastewater treatment plants (WWTPs) is well documented [2,9,10]. The presence of ZnO-NPs also hinders the degradation and removal of phosphorous and nitrogen in wastewater biofilms and activated sludge [8,11,12]. Understanding the fate and behaviour of ZnO-NPs once they are released, transferred, or interact with certain environmental factors is critical for evaluating their potential risks. Environmental factors, such as ionic strength, natural organic substances, pH, light, and polymeric substances, can significantly influence the colloidal stability and toxicity of ZnO-NPs [13,14]. For instance, the presence of hexabromocyclododecane or polybrominated diphenyl ethers as organic pollutants in water systems can alter the size and surface charge of ZnO-NPs [15,16]. Alterations in the surface potential and dispersion of humic acid-adsorbed/coated ZnO-NPs in aquatic environments have been reported [17]. Studies have also noted the unstable (aggregation) behaviour of nanoparticles (such as ZnO-NPs) in salt water (high ionic strength) due to a reduction in/compression of the thickness of the electrical double layer followed by a reduction in the energy barrier [18,19,20]. Normally, organic contaminants tend to interact in water media due to their hydrophobic nature, but they are more likely to interact and sorb onto the surface of ZnO-NPs (due to their high surface area). The possible interactions include van der Waals, electrostatic, hydrophobic, and π-π interactions followed by ligand exchange, hydrogen bonding, and molecular bridging effects [21,22,23]. These interactions can greatly alter the fate and behaviour of contaminants through aggregation, dispersion, surface charge alteration, surface coating/adsorption, and changes in crystallinity and purification.

Studies have also reported the interaction mechanisms of engineered nanoparticles with endocrine disrupting chemicals, such as polybrominated diphenyl ethers [16,22,24,25] and hexabromocyclododecane [15] as emerging environmental chemicals. Per- and polyfluoroalkyl substances (PFAS) are a class of endocrine disrupting compounds that may disrupt human thyroid hormone systems with possible negative impacts on pregnancy followed by fetal–child development [26]. The presence of such endocrine disrupting compounds could also influence the behaviour of ZnO-NPs. This study examined the changes in the behaviour of ZnO-NPs during interaction with PFOA, a representative PFAS, under various environmentally relevant conditions in water media.

PFOA is largely used in water-resistant products, such as carpets, paints and coatings, waterproof clothing, firefighting foams, nonstick cookware, and hydraulic fluids [27]. Concerns are rising regarding the release of PFOA into the environment due to its bioaccumulative and toxic nature [27,28,29]. PFOA has been identified in the influent and effluent of WWTPs and biosolids [29,30,31]. PFOA concentrations in influents of various WWTPs in Canada, Spain, China, Singapore, and North America have been reported to range from 2.2 ng/L to 6.6 × 10^4^ ng/L, while they ranged from 1.3 ng/L to 1.6 × 10^5^ ng/L in effluents [29]. Similarly, a range of concentrations of PFOA in the biosolids of many WWTPs in North America, Switzerland, Spain, Australia, China, Kenya, Canada, Singapore, and Finland has been documented, from 0.03 ng/g to 158 ng/g [29].

The national loads of PFOA in the effluents of 14 WWTPs in Australia were estimated to be 65 kg/year and 2 kg/year for biosolids [29]. Various drinking water sources in many countries now contain PFOA [32], including Australia (0–9.7 ng/L), Brazil (0.81–2.8 ng/L), India (<0.005–2 ng/L), China (<0.1–45.9 ng/L), Germany (<10–68 ng/L), Japan (2.3–84 ng/L), and the USA (<5–30 ng/L). Several hundred nanograms per liter of PFOA have been reported in surface waters [33]. Sediments and biota can also contain PFOA at concentrations ranging from pg/g to a few ng/g [32,34]. The maximum concentrations of PFAS, including PFOA, in marine (onshore) waters have been detected (up to 58 ng/L), while 0.11 ng/L PFOA have been found in offshore waters [32]. PFOA concentrations ranging from 1 to 13 ng/L in Palermo’s (Sicily) coastline seawaters have also been detected and reported [35]. 

The adsorption of PFOA may lead to an accumulation of organic compounds on the surface of ZnO-NPs when these nanoparticles are released into natural waters (e.g., from sunscreen). In this study, PFOA and its interactions with ZnO-NPs were investigated, as well as the factors enabling these interactions. In particular, the physico-chemical properties, colloidal stability, particle size and surface charge alterations of the ZnO-NPs before and after interaction with PFOA under various simulated water conditions were assessed, such as in the presence of electrolytes (sodium chloride, NaCl, and calcium chloride, CaCl_2_) and HA. The concentration of PFOA in natural waters is much lower than some of the concentrations considered in this study, which enabled characterization of the changes in ZnO-NPs. The findings of this study will be useful for assessing the influence of environmental water conditions on the exposure of ZnO-NPs and their co-contaminants.

## 2. Materials and Methods

### 2.1. Materials and Chemicals

The characteristics of the purchased ZnO-NPs, HA, and electrolytes have been reported in our previous paper [19]. The ZnO-NPs < 100 nm particle size, 544906-50G, were purchased from Sigma Aldrich Australia. The HA was purchased from Sigma Aldrich (53680-50G, humic acid technical) Australia. Most of the nanoparticles were <100 nm in size, and some were larger than 100 nm due to aggregation. PFOA was purchased from Sigma–Aldrich (C_8_HF_15_O_2_, molecular weight: 414.07 g/mol, and purity: 96%), Australia, and subjected to characterization for the purposes of this study.

### 2.2. Interaction between PFOA and ZnO-NPs

The ZnO-NPs stock suspension was prepared by adding 0.1 g of nanoparticles to 1 L of Milli-Q water, followed by sonication for 10 min. Various concentrations of PFOA, from 0, 0.5, 1, 10, 50, 100, 200, and 500 µg/L to 1, 5, 10, and 50 mg/L, were prepared in Milli-Q water containing the ZnO-NPs (0.1 g/L) suspension. The higher concentrations of PFOA aimed to amplify the effects of interaction to be detectable by the Zetasizer and particle size analyzer and help to understand the mechanism of interaction. Such alteration effects are difficult to detect with PFOA at environmentally relevant concentrations due to limitations in characterization techniques. The nanoparticles were analyzed (particle size, zeta potential, dissolution, adsorption, XRD, Raman spectroscopy, FTIR, and TEM) before and after interaction with PFOA in solution at various time intervals, such as after 1 day, 1 week, 2 weeks, and 3 weeks of interaction, to assess the changes in the behaviour of the interacting nanoparticles in comparison with that of the pure nanoparticles.

### 2.3. Influence of Electrolytes on PFOA and ZnO-NPs’ Interaction

The influence of mono- and divalent electrolytes (such as NaCl and CaCl_2_) on the behaviour of ZnO-NPs was observed in the presence of various concentrations of PFOA (such as 10 µg/L and 500 µg/L) as an organic pollutant. The ZnO-NP stock suspension was prepared by adding 0.1 g of nanoparticles to 1 L of Milli-Q water, followed by sonication for 10 min. PFOA concentrations (10 and 500 µg/L) were also prepared in Milli-Q water containing the ZnO-NPs (0.1 g/L) suspension. Fixed concentrations (i.e., 5 mM) of monovalent and divalent salts (NaCl and CaCl_2_) were used to investigate the effect of electrolytes on the stability of ZnO-NPs alone and in the presence of PFOA. The changes in the particle size and zeta potential of ZnO-NPs after various time intervals, i.e., after 1 day, 1 week, 2 weeks, and 3 weeks of interaction, including PFOA adsorption on the surface of ZnO-NPs in the presence of salts, are examined, which are presented and discussed in Section 3.2.

### 2.4. Influence of HA on PFOA and ZnO-NPs’ Interaction

The effect of HA, a natural organic substance, on the size and zeta potential of the ZnO-NPs was investigated with and without 10 and 500 µg/L PFOA. The HA powder was dissolved in 0.1 M NaOH solution to prepare the stock solution. Various concentrations of HA with ZnO-NPs (0.1 g/L) suspensions in the absence and presence of PFOA were prepared. Changes in the size, shape, and charge of nanoparticles with and without PFOA and HA were investigated. The amount of PFOA adsorbed onto the surface of the ZnO-NPs was also analyzed after 1 day and 2 weeks of interaction. The pH of all the samples was maintained at 7 by using a buffer solution (thermos scientific buffer solution pH 7, USA) composed of potassium dihydrogen phosphate. The prepared suspensions were analyzed using a Malvern Panalytical Zetasizer, UK. A Zetasizer was used at room temperature (i.e., 20 °C) using a disposable folded capillary cell. The nanoparticles in the suspension were directly dropped onto TEM Cu grids to exam any morphological changes in the ZnO-NPs (such as ZnO-NPs in water suspensions and in the presence of PFOA, HA) for TEM analysis. ZnO-NPs were also obtained for FTIR, XRD, and Raman analysis by separating them from the suspension using a high speed centrifuge.

### 2.5. Influence of Electrolytes and HA Together on PFOA and ZnO-NPs’ Interaction

The influence of HA and electrolytes together on the size and zeta potential of the ZnO-NPs was investigated with and without 10 and 500 µg/L PFOA and fixed concentration (5 mM) of various electrolytes (such as NaCl and CaCl_2_). Various concentrations of HA with ZnO-NPs (0.1 g/L) suspensions in the absence and presence of PFOA and electrolytes were prepared. Changes in the size, shape, and charge on the surface of nanoparticles with and without PFOA, electrolytes, and HA were investigated. The amount of PFOA adsorbed onto the surface of the ZnO-NPs was also analyzed after 1 day and 2 weeks of interaction, with results discussed in Section 3.4.

### 2.6. Characterization Methods

FE-SEM (Zeiss Sigma VP Field Emission Scanning Electron Microscope, Germany) was performed at 15 kV after sputter coating (10 nm platinum layer), and TEM (JEM 2100 LaB6 High Resolution Transmission Electron Microscope, Japan) was performed at 200 kV to investigate the morphology. Copper grids (Lacey carbon film, 300 mesh) for TEM imaging were obtained from PST (ProSciTech), Australia. The micromeritics TriStar ׀׀ (Microtrac, USA) and an XRD system (Empyrean Malvern PANalytical, Malver, UK) were used to calculate the nanoparticle surface area, pore size distribution, and phase-dimensional identification. FTIR (Agilent Technologies, Cary 600 Series) was used to determine the functional groups present before and after interactions (wavenumber range: 400−4000 cm^−1^ and number of scans: 16). Raman spectra were recorded using a WITec confocal Raman microscope (Alpha 300 RS, Germany) equipped with a 532 nm laser diode (<30 mW) under an objective lens (×100 or others) at room temperature.

A Malvern Panalytical Zetasizer was used to determine the changes in charge and size of the ZnO-NPs alone and in the presence of PFOA, HA, and different electrolytes in a pure water state (Milli-Q water). Disposable folded capillary zeta cells (DTS1070) for dynamic light scattering were obtained from Malvern Instruments Ltd. in Malvern, UK. An inductively coupled plasma/optical emission spectroscope (ICP–OES, PerkinElmer’s NexION 350x) was used to determine the concentrations of dissolved zinc in the water samples before and after interactions with PFOA. An Agilent liquid chromatography–mass spectrometry (LC–MS) was employed to determine the adsorbed amount of PFOA before and after interaction.

## 3. Results and Discussion

### 3.1. Interaction between PFOA and ZnO-NPs

**Figure 1:**
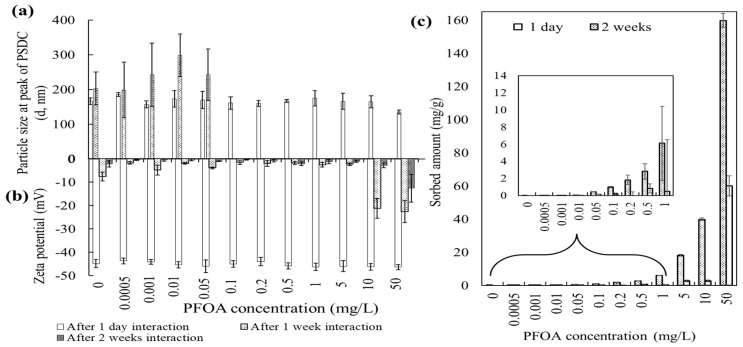
The analysis of particle size (**a**), zeta potential (**b**), and sorption of PFOA by ZnO-NPs (**c**), after ZnO-NPs interaction with PFOA at different times. Zeta potential values are minus.

Particle size: Alterations in the particle size and surface charge of the ZnO-NPs were observed following interaction with various concentrations of PFOA. With the addition of various concentrations of PFOA to the ZnO-NPs (after 1 day of interaction), the particle size of the ZnO-NPs at the peak of the particle size distribution curve (PSDC) remained between 157 and 186 nm, and the particle size ranged from 106–955 nm (Figure 1a and Figure S1a). The slight variations in the particle size at the peak of the PSDC and within the particle size ranges after 1 day of interaction may be due to less interaction time between the nanoparticles and the organic compound (PFOA). It is suggested that interactions between ZnO-NPs and PFOA as organic pollutants could be time-dependent after their coexistence was ascertained.

A significant increase in the particle size of the ZnO-NPs was observed after 1 week of interaction with PFOA. The size of the ZnO-NPs at the peak of PSDC increased from 166 nm to 203 nm, and similar trends were observed in the presence of PFOA up to 50 µg/L PFOA (Figure 1a and Figure S1b). However, the size ranges of ZnO-NPs in the presence of PFOA from 100 µg/L to 50 mg/L were not measurable via a Zetasizer, with a maximum size ranging from 0.3 nm to 10 microns. It could be assumed that the size of the ZnO-NPs after 1 week of interaction with PFOA (from 100 µg/L to 50 mg/L) is more than 10 microns. The alteration in the size distribution of the ZnO-NPs was influenced by the presence of PFOA through aggregation and the magnitude of the surface coating/adsorption of organic substances [14,22,36]. Overall, an increase in the particle size of the ZnO-NPs was observed alone and in the presence of PFOA from 1 day to 3 weeks of interaction. This increase might be due to the presence of large (agglomerated) and/or sedimenting particles resulting from particle–particle interactions, electrostatic interactions, and hydrophobic interactions. The ZnO-NPs were monodispersed (particles of uniform size) from the time of the nanoparticle suspension preparation to a few days later. However, after 1 week of interaction, nonuniform (polydisperse) behaviour of the nanoparticles was observed. Overall, particles from 1 week to 3 weeks were found in their polydispersed form, which was also reflected by their surface charge.

Zeta potential: The zeta potential (Figure 1b) of the ZnO-NPs did not significantly change after 1 day of interaction with the addition of different concentrations of PFOA, while a significant decreasing trend was observed for samples after 1 and 2 weeks of interaction. The alterations in the charge potentials suggested that PFOA coating/adsorption on the surface of the ZnO-NPs decreased in the magnitude of surface charge values. The zeta potential values also suggested that the ZnO-NPs became less stable in solution and tended to agglomerate (increasing size, Figure 1a and Figure S1b) with increasing concentrations of PFOA. This aggregation might be attributed to the higher molecular weight and greater surface coating of PFOA on the ZnO-NPs via hydrophobic and van der Waals interactions [36,37,38]. The hydrophobic tail of PFOA has a decreased tendency to interact with water molecules, while the hydrophilicity and charged head of PFOA could lead to a greater tendency to attach to the ZnO-NP surface. The aggregation suggested the unstable nature of the ZnO-NPs after interactions with PFOA [15,16].

Adsorption: The adsorption analysis of PFOA in solution confirmed the interaction between ZnO-NPs and PFOA after 1 day and 2 weeks of interaction. Figure 1c shows the increased adsorption of PFOA molecules on the surface of the ZnO-NPs after 2 weeks of interaction compared to after 1 day. Various interaction mechanisms could be involved in the process of PFOA adsorbing onto the surface of ZnO-NPs, which increased with time. An increase in the particle size and an overall decrease in the net charge potential of the surface nanoparticles were observed (Figure 1a,b). Once the ZnO-NPs are in water, the formation of hydroxide layers ($Zn{\left(OH\right)}_{\left(aq\right)}^{+})$ on the surface of the nanoparticles due to hydrolysis is a common process, as water molecules can be adsorbed (both chemically and physically) onto the surface of the particles [22,36,39]. This resulted in the formation of a quantity of positive charges on the surface of the nanoparticles, attracting PFOA^−^ molecules to adsorb to the surface of the ZnO-NPs by electrostatic interactions. After that, the hydrophobic tail of PFOA^−^ combined on the surface of the ZnO-NPs adsorbed the free PFOA^−^ molecule in the solution by hydrophobic interactions (hydrophobic PFOA molecules tend to accumulate more on the surface of the ZnO-NPs in aqueous media) [40] and further increased the adsorption amount of PFOA after 2 weeks’ time interval compared to that after 1 day. This can also be corroborated by the decreased surface charge of the ZnO-NPs and the increase in particle size (Figure 1a,b).

Dissolution: The dissolution of ZnO-NPs in terms of dissolved zinc (mg/L) after 1 day and 2 weeks of interaction with PFOA was determined via ICP–OES (Figure S3). The concentration of dissolved zinc in the 0 µg/L sample (i.e., ZnO-NPs in buffered water) slightly increased after 2 weeks of interaction compared to that in the 1 day interaction. The dissolved zinc concentration increased in the presence of PFOA, which further decreased after 2 weeks of interaction compared to that for 1 day. This could be due to the aggregation/agglomeration and settlement of particles in the test tube, where coprecipitation occurred and reduced the dissolved amount of zinc in solution. The dissolution rate is often claimed to be directly proportional to the specific surface area of the material, meaning that the smaller the particles are, the faster dissolution occurs [41]. Agglomeration has been proposed as a rationale for slow dissolution [42]. Moreover, aggregation/agglomeration may have resulted in decreased dissolution [42] as the particle size increased and the settlement of particles at the bottom of the test tube was observed. The surface properties of ZnO are quite complicated due to the presence of polar and nonpolar crystallographic planes [43]. The interaction of PFOA molecules with nonpolar planes leads to agglomeration and less dissolution. A similar dissolution trend was observed in the presence of electrolytes and HA, as shown in the next sections.

**Figure 2:**
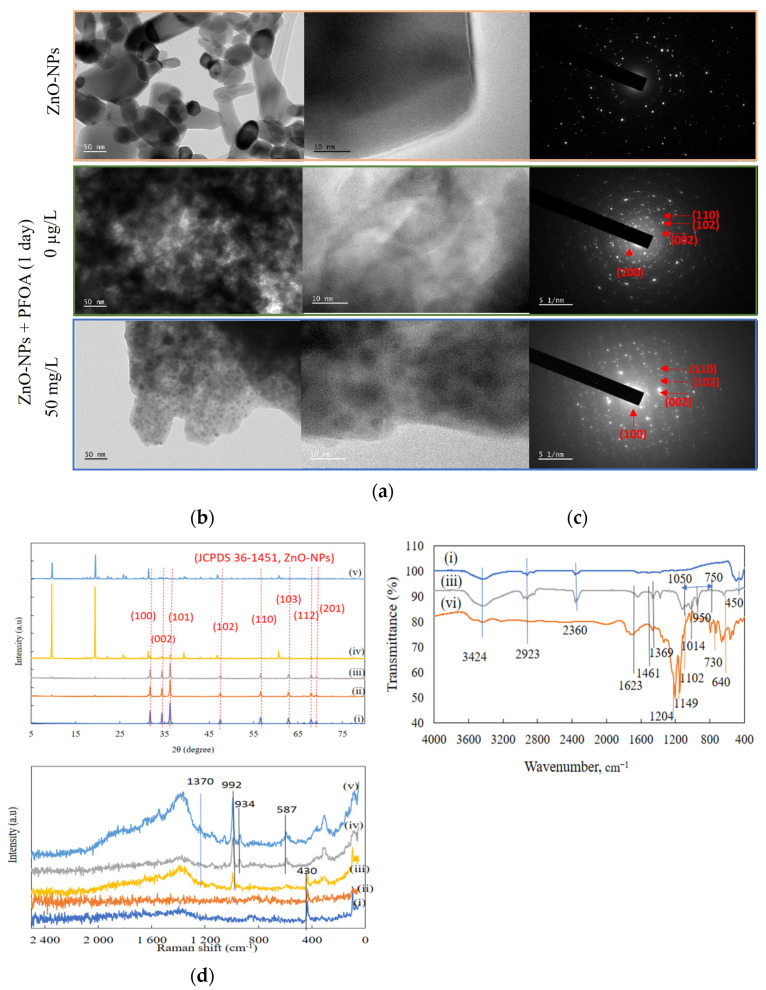
The characterization analysis for ZnO-NPs before and after interaction with PFOA (50 mg/L): (**a**) TEM; (**b**) XRD; (**c**) FTIR; (**d**) Raman; samples include (i) ZnO -NPs; (ii) ZnO-NPs in buffered Milli-Q water (pH 7) after 1 day; (iii) ZnO-NPs in buffered 50 mg/L PFOA (pH 7) after 1 day; (iv) ZnO-NPs in buffered Mili-Q water (pH 7) after 3 weeks; (v) ZnO-NPs in 50 mg/L PFOA (pH 7) after 3 weeks; (vi) PFOA powder.

TEM, XRD, FTIR, and Raman analysis: Alterations in the morphology of ZnO-NPs were observed after 1 day of interaction with PFOA using TEM (Figure 2a). The original (virgin) ZnO-NPs (Figure 2a) were in an agglomerated/spongy form with a particle size ≤ 100 nm. Both rod and spherical morphologies were observed. The lattice pattern and glittering spots/rings suggest the crystalline structure of the ZnO-NPs [15,44]. The dissolved zinc concentrations in Milli-Q water at various pH values for the same batch of ZnO-NPs were reported in our previous research [15], which indicated minimal dissolution at pH ≥ 7. The changes in crystallinity after the interaction of ZnO-NPs with water were also examined using TEM analysis. TEM showed a diffraction pattern (Figure 2a) and the crystalline/lattice arrangement of the original ZnO-NPs [15,44]. The mixture was dull and cloudy after interacting with buffer containing Milli-Q water. A more disordered/amorphous structure of the ZnO-NPs (Figure 2a) was observed after interaction with PFOA. Similarly, compared with those of the pure powder, the diffuse and cloudy bright spots (Figure 2a) represented the impure morphology of the ZnO-NPs after interaction with PFOA. The elemental composition obtained from TEM analysis is shown in Figure S2a. The structural parameters, such as lattice spacing and the crystallite size of the ZnO-NPs after 1 day of interactions (ZnO-NPs in Milli-Q water and ZnO-NPs + 50 mg/L PFOA) were also measured using TEM analysis (Figure S2b). Lattice spacing of 0.26 nm was found in the lattice structure of ZnO-NPs. However, the crystallite size ranging from 4.29 nm to 8.39 nm were observed after 1 day of ZnO-NPs interactions with 50 mg/L PFOA (Figure S2b).

X-ray diffraction (XRD) analysis of powder ZnO-NPs and ZnO-NPs in buffered Milli-Q water and with 50 mg/L PFOA after 1 day of interaction is shown in Figure 2b. Sharp peaks at 2θ values of 31.84°, 34.6°, and 36.5° are observed for the ZnO-NPs, which represent the (hexagonal wurtzite) crystal structure of the nanoparticles with three perfect alignments: (1 0 0), (0 0 2), and (1 0 1). These alignments match the defined standard powder diffraction (JCPDS, No. 36-1451) [22,45]. This result revealed no change in the crystal phase of the ZnO-NPs after 1 day of interaction. However, after 3 weeks of interaction (Figure 2b), the intensities of the peak alignments at (1 0 0), (0 0 2), and (1 0 1) were suppressed. Two new peaks at 2θ values of 9.62° and 19.35° were observed, which could be due to the formation of new compounds, such as zinc hydroxide dihydrate Zn_5_(OH)_10_·2H_2_O [46] and zinc phosphate nanocrystalline materials [47], respectively. The intensities of the peaks at 2θ values of 9.62° and 19.35° were lower for the ZnO-NP sample with 50 mg/L PFOA than for the ZnO-NP sample in buffered water, which could be due to the coating of PFOA molecules onto the surface of the ZnO-NPs (Figure 2b).

FTIR analyses of the ZnO-NPs, PFOA, and ZnO + PFOA were also conducted (Figure 2c). The peak at 450 cm^−1^ suggested the presence of Zn–O [15,48], which is in the range of metal oxides (400–600 cm^−1^) [15,49]. A very low-intensity peak at 450 cm^−1^ was also observed for the sample of ZnO-NPs + PFOA after interaction, indicating the presence of ZnO-NPs after interaction with PFOA molecules. The peak at 640 cm^−1^ is due to characteristic bands of organic halogen compounds (such as C–F stretching) [50], which were identified in both PFOA and ZnO-NPs + PFOA after interacting with PFOA. The vibrational peaks at 1102, 1149, and 1204 cm^−1^ may be due to C–F stretching, with the peak at 1102 cm^−1^ being identified in the spectrum of the ZnO-NPs after interaction with PFOA. C–H stretching, O–H stretching, and C=O stretching was also identified at 1369, 1461, and 1623 cm^−1^, respectively [50,51]. These peaks were identified in both the PFOA and ZnO + PFOA samples, indicating the link between PFOA and ZnO-NPs after their interaction. The peak at 2360 cm^−1^ could be due to carbon dioxide from the atmosphere. The peak at 3424 cm^−1^ is due to stretching of the water band [15,50]. The peak detected between 750 and 1050 cm^−1^, at 950 cm^−1^, could be due to K-potassium and P-phosphorous (from a buffer solution used to maintain pH 7) stretching with O and C in the ZnO-NPs after interaction with PFOA [50]. The FTIR results revealed the presence of bonds in the ZnO-NPs after interaction with PFOA, suggesting that there was an association between PFOA and the ZnO-NPs. This is consistent with the elemental analysis results indicating the presence of F and C in the ZnO-NPs.

The Raman spectrum (Figure 2d) also confirmed the presence of ZnO-NPs (such as at 430 cm^−1^) in the pure ZnO-NPs samples in buffered water and in the presence of PFOA after 1 day of interaction. After 3 weeks of interaction, new peaks at 587, 934, 992, and 1370 cm^−1^ were observed for both samples (such as ZnO-NPs in water and with PFOA) including ZnO-NPs with PFOA after 1 day of interaction (Figure 2d). Zn−O stretches fall in the region between 350 and 600 cm^−1^. The spectral range of Zn−OH bonds (which are also called OH linkages) is between 600 and 1200 cm^−1^. The asymmetric stretches (with a high infrared intensity) are in the range 470−550 cm^−1^, whereas the symmetric stretches were observed at 368 and 382 cm^−1^ in the Raman spectrum. Bands below 350 cm^−1^, as observed in the Raman spectrum, are attributed to lower-energy lattice modes [46].

### 3.2. Influence of Electrolytes on PFOA and ZnO-NPs’ Interaction

The salinity of surface water and groundwater can vary considerably. Salinity is one of the most significant abiotic factors affecting the growth, metabolism, immunity, and survival of aquatic species in farming environments. Due to global climate change, evaporation of seawater, variations in local rainfall, and the whereabouts of ocean currents, environmental salinity in coastal areas alters frequently and violently. Under environmental stresses, physiological mechanisms are adaptively modulated to sustain body homeostasis, which can further impact the normal biological functions, comprising the immunity of the aquatic species [52]. The presence of salts could influence the interaction between ZnO-NPs and coexisting contaminants, which determines the environmental fate of ZnO-NPs. The ionic strength, pH, and other organic materials present in the solution could influence the surface charge and stabilization of ZnO-NPs. Fixed concentrations (i.e., 5 mM) of monovalent and divalent salts (NaCl and CaCl_2_) were used to investigate the effect of electrolytes on the stability of ZnO-NPs alone and in the presence of PFOA. The changes in the particle size and zeta potential of ZnO-NPs after various time intervals, i.e., after 1 day, 1 week, 2 weeks, and 3 weeks of interaction, are presented and discussed below.

**Figure 3:**
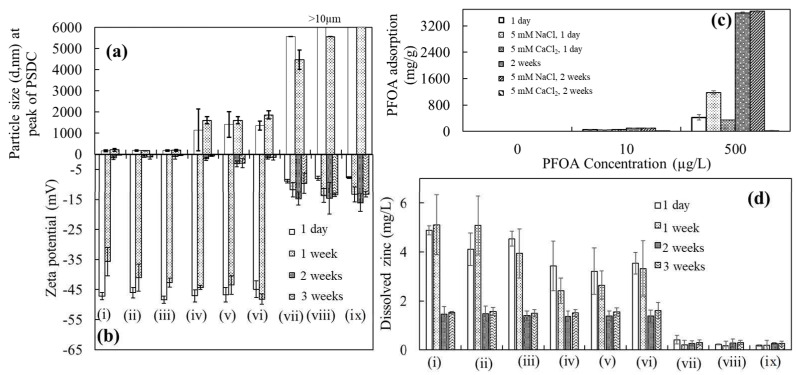
Analysis of particle size (**a**) and zeta potential (**b**) for ZnO-NPs after interaction with PFOA and electrolytes; analysis of PFOA sorption (**c**) and dissolved Zn (**d**) in the solution after interaction; samples include: (i) ZnO-NPs; (ii) ZnO-NPs in 10 µg/L PFOA; (iii) ZnO-NPs in 500 µg/L PFOA; (iv) ZnO-NPs in 5 mM NaCl; (v) ZnO-NPs in 10 µg/L PFOA and 5 mM NaCl; (vi) ZnO-NPs in 500 µg/L PFOA and 5 mM NaCl; (vii) ZnO-NPs in 5 mM CaCl_2_; (viii) ZnO-NPs in 10 µg/L PFOA and 5 mM CaCl_2_; (ix) ZnO-NPs in 500 µg/L PFOA and 5 mM CaCl_2_. Zeta potential values are minus.

Particle size: Alterations in the size of ZnO-NPs were observed alone (i.e., ZnO-NPs in buffered Milli-Q water and PFOA) and in the presence of salts with PFOA after 1 day, 1 week, 2 weeks, and 3 weeks of interaction (Figure 3a and Figure S4). The particle size of the ZnO-NPs in buffered water increased after 1 week of interaction compared to that after 1 day, e.g., 162.4 nm after 1 day to 206 nm after 1 week of interaction; these findings are consistent with the results shown in Figure 1a (166 nm after 1 day and 203 nm after 1 week of interaction) and Figure S1a,b. Similarly, an increase in the size of the ZnO-NPs was observed in the presence of 10 and 500 µg/L PFOA. A similar effect on the correlation between particle size and the surface charge of ZnO-NPs was observed for some samples, such as ZnO-NPs with 10 µg/L PFOA and ZnO-NPs with 500 µg/L PFOA (Figure 3a,b).

For instance, in Figure 1a, the size at the peak of the PSDC of ZnO-NPs with 10 µg/L PFOA is 173 nm after 1 day and 298.5 nm after 1 week, with a surface charge of −45.4 mV after 1 day and −1.9 mV after 1 week. The size of ZnO-NPs was also increased affecting the surface charge of ZnO-NPs at the same concentration (Figure 3a,b); the larger the particle size, the greater the decrease in the magnitude of the surface charge values and vice versa. A similar observation (such as a drop in magnitude of surface change with the increase in the particle size) with surface charge was observed for different batches of samples of the same concentration (such as ZnO + 10 and 500 µg/L PFOA, Figure 1a,b and Figure 3a,b). After 2 and 3 weeks, the instability trend was similar, with the particle size being outside the machine range. This result showed that, initially, the stability of ZnO-NPs could vary based on Brownian motion (the random movement of particles due to bombardment by the solvent molecules that surround them). Normally, dynamic light scattering involves the measurement of particles suspended within a liquid and their shape. If the shape of a particle changes in a way that affects the diffusion speed, then the hydrodynamic size and surface charge may also change. This trend remained consistent with these concentrations (such as ZnO + 10 and 500 µg/L PFOA) in the next sections.

An increase in the particle size with a decreased surface charge was also observed in the presence of 5 mM NaCl with ZnO-NPs and with ZnO-NPs and PFOA after various time intervals (Figure 3a,b). The ions in the medium and the total ionic concentration may affect the particle diffusion speed by altering the thickness of the electric double layer (the Debye length, K^−1^). The resulting extended double layer of ions around the particles due to electrostatic interactions leads to a reduction in the diffusion speed and results in a larger, apparent hydrodynamic diameter. However, the presence of hydrophobic PFOA molecules, which can accumulate more easily around the surface of nanoparticles due to their hydrophobic nature than when suspended in water, may also alter the size and charge of the nanoparticles.

Generally, the stability of nanoparticles in the presence of electrolytes strongly depends on the capping agents used for stabilization [53,54]. Variations in the size of the ZnO-NPs with the co-occurrence of PFOA and electrolytes could be linked to adsorption, electrostatic interactions, hydrogen bonding, van der Waals effects, and cation bridging (Schematic 7). For instance, in the case of 5 mM NaCl, few alterations in the particle size were observed at the peak of the PSDC (Figure 3a). However, the presence of 5 mM monovalent salts (NaCl) balanced the net electrostatic interactions between highly electronegative fluorine atoms and salt due to charge screening/shielding effects and London interactions [54,55].

The influence of divalent cations on the size of ZnO-NPs was different in the presence of PFOA. Alterations in the size and surface charge of the ZnO-NPs were observed by interacting the ZnO-NPs with 5 mM CaCl_2_ alone or in the presence of PFOA after 1 day, 1 week, 2 weeks, and 3 weeks of interaction (Figure 3 and Figure S4). This increase in the size of the ZnO-NPs could be due to the accumulation of divalent cations, which results in a decrease in the diffusion speed and generation of larger particles compared to those of NaCl. It could also be expected that in the presence of 5 mM CaCl_2_, the electrostatic repulsion between the negatively charged ZnO-NPs surface and PFOA molecules (which have a negative charge due to the anionic nature and high electronegativity) was reduced because of bridging interactions between the negatively charged surfaces of ZnO-NPs, divalent cations, and PFOA molecules. Consequently, the gathering of positively charged divalent cations enhanced the nanoparticle size due to bridging effects and made the nanoparticles more unstable, leading to agglomeration.

Cations, such as Ca^2+^, could be the cause of the bridging phenomenon between carboxyl groups. Consequently, the adsorption of PFOA may be hindered in certain aqueous environments enriched with the aforementioned cations due to the decrease in electrostatic interactions between PFOA and the protonated surface [56,57,58]. The same behaviour was observed for the ZnO-NPs in the following experiments.

Zeta potential: The zeta potentials of the ZnO-NPs (0.1 g/L nanoparticle dispersion, experimental batch one) were −47.2 and −35.7. −1.1, and −0.2 mV after 1 day, 1 week, 2 weeks, and 3 weeks of interaction, respectively (Figure 3b). The decrease in the surface charge of the ZnO-NPs could be caused by the aggregation of nanoparticles resulting from van der Waals forces, hydrogen bonding, and hydrophobic interactions based on the aging factor. A similar decreasing trend in the magnitude of the surface charge was observed in the presence of PFOA (Figure 3b). The presence of mono- and divalent salts also altered the surface charge of the ZnO-NPs after various durations of interaction with PFOA.

The presence of 5 mM NaCl did not significantly change the zeta potential of the nanoparticles after 1 day of interaction alone or in the presence of PFOA (Figure 3b). It could be argued that the large extent of PFOA adsorption on the surface of nanoparticles balanced the overall electrostatic interaction forces between PFOA molecules and the monovalent salts based on shielding effects and London interactions [54,55]. However, the surface charge of the ZnO-NPs (in the presence of 5 mM NaCl) decreased to −0.5 mV after 3 weeks of interaction. This may indicate that aging affects the surface charge of nanoparticles, allowing more attachment of monovalent cations to the negatively charged surface of ZnO-NPs via electrostatic forces of attraction and van der Waals interactions. However, the same decreasing trend after various numbers of interactions was also observed in the presence of PFOA molecules (Figure 3b).

Similarly, a decrease in the magnitude of the zeta potential of the ZnO-NPs was observed in the presence of divalent cations (5 mM CaCl_2_) alone and in the presence of 5 mM CaCl_2_ at various concentrations (such as 10 and 500 µg/L) of PFOA (Figure 3b). The electrostatic repulsion between the ZnO-NP (negatively charged) surface and PFOA molecules was reduced in the presence of 5 mM CaCl_2_. The gathering of positively charged ions on the surface was responsible for the decrease in zeta potential (less negative zeta potential) (Figure 3b), which increased the particle size due to bridging effects and increased instability, leading to agglomeration (Figure 3a and Figure S4). The aggregation behaviour of ZnO-NPs in the presence of salts suggested that aging during the interaction of ZnO-NPs with salts affects the particle diffusion speed by changing the thickness of the Debye length due to the gathering of ions, resulting in agglomeration alone and in the presence of organic pollutants.

Adsorption: PFOA was analyzed to investigate the effects of salts (such as 5 mM NaCl and 5 mM CaCl_2_) on the adsorption (interaction) of PFOA (10 and 500 µg/L) on ZnO-NPs after 1 day and 2 weeks of interaction (Figure 3c). Increased adsorption (interaction) of PFOA molecules was identified with increasing concentration and interaction time (more sorption after 2 weeks than after 1 day). A similar trend with increased adsorption of PFOA was observed in the presence of 5 mM NaCl. A possible explanation for this result could be that the increase in the ionic strength of monovalent ions (Na^+^) due to NaCl caused an increase in electrostatic attraction between the negatively charged ZnO-NP surface and the negatively charged PFOA molecules due to the presence of monovalent ions (Na^+^) in between serving as a bridging carrier to support bridging interactions. However, less adsorption of PFOA on ZnO-NPs was observed with a 5 mM CaCl_2_ concentration in the solution for both time intervals. Both CaCl_2_ and NaCl affected the adsorption of PFOA on ZnO-NPs, potentially due to the electrostatic force of attraction. However, in the case of CaCl_2_, the bridging effect of divalent (Ca^2+^) cations between ZnO-NPs and PFOA may further lead to a reduction in PFOA adsorption on the ZnO-NPs [57,59]. This finding is consistent with one study showing that the adsorption of PFOA decreases with increasing ionic strength [57].

Dissolution: The dissolved zinc (mg/L) in the ZnO-NPs in buffered water and after the interaction of PFOA with the ZnO-NPs in the presence of salts (such as 5 mM NaCl and 5 mM CaCl_2_) were measured using ICP–OES (Figure 3d). The particle size increased due to agglomeration and sedimentation after several weeks of interaction, decreasing the specific surface area and resulting in restrained dissolution. However, in the case of CaCl_2_, less dissolution was measured than in all the other samples, which may be related to more agglomeration due to the bridging effect of Ca^2+^. The smaller the size of the ZnO-NPs, the more easily dissolution occurred compared to the dissolution of larger particles [18]. The attachment and penetration of nanoparticles inside the pores of low-density polyethylene tubes cannot be ignored.

### 3.3. Influence of HA on PFOA and ZnO-NPs’ Interaction

**Figure 4:**
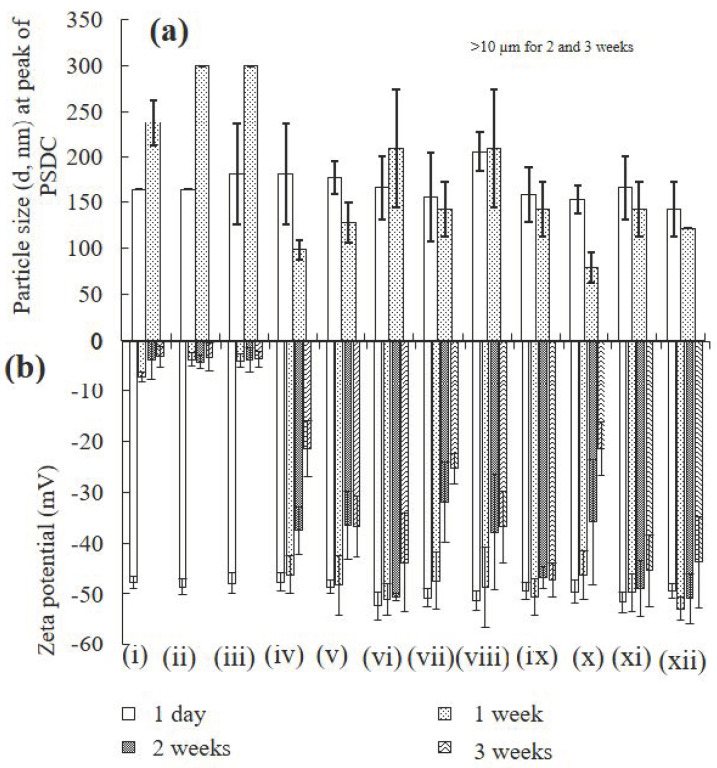

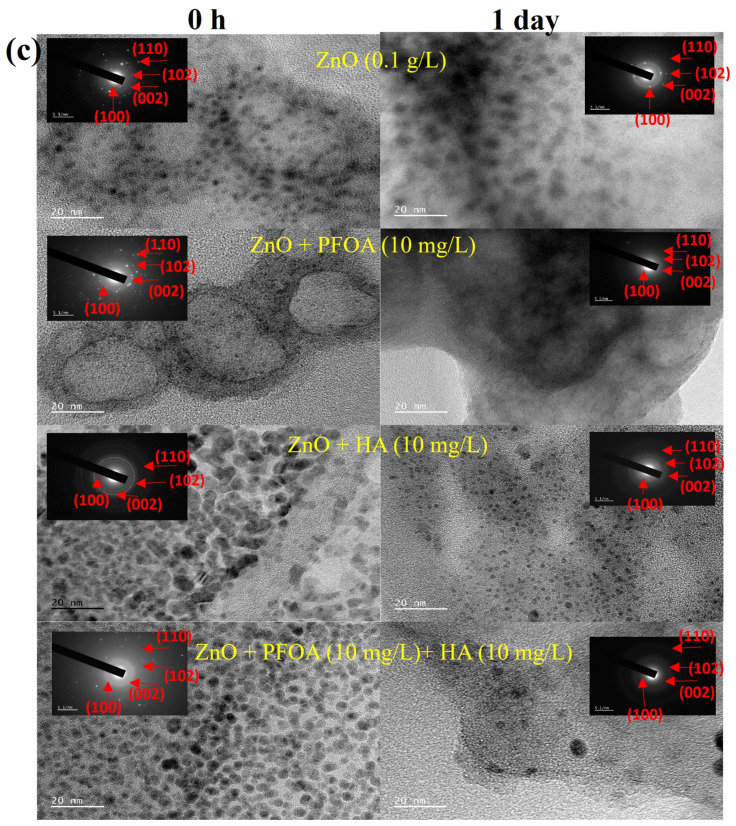
The analysis for ZnO-NPs after interaction with PFOA and HA; particle size (**a**), zeta potential (**b**), and TEM (**c**); samples include: ZnO-NPs (i), ZnO-NPs in 10 µg/L PFOA (ii) and 500 µg/L PFOA (iii); ZnO-NPs in 1 (iv), 5 (v) and 10 (vi) mg/L HA; ZnO-NPs in 10 µg/L PFOA and 1 (vii), 5 (viii) and 10 (ix) mg/L HA; ZnO-NPs in 500 µg/L PFOA and 1 (x), 5 (xi), and 10 (xii) mg/L HA. Zeta potential values are minus. The scale bar of all SAED images is 5 1/nm.

Particle size: The sizes of the ZnO-NPs in buffered water, treated with various concentrations of PFOA (such as 10 or 500 µg/L), treated with various concentrations of HA (1, 5, or 10 mg/L), and mixed with each of the other substances, were analyzed after 1 day, 1 week, 2 weeks, and 3 weeks of interaction at pH 7 (Figure 4a and Figure S5). Overall, an increase in the particle size of the ZnO-NPs was observed alone and in the presence of PFOA from 1 day to 3 weeks of interaction. This increase might be due to the presence of large (agglomerated) and/or sedimenting particles resulting from particle–particle interactions, electrostatic interactions, and hydrophobic interactions. The ZnO-NPs were monodispersed (particles of uniform size) from the time of nanoparticle suspension preparation to a few days. However, after 1 week of interaction, nonuniform (polydisperse) behaviour of the nanoparticles was observed. Overall, particles with sizes ranging from 1 week to 3 weeks were obtained in their polydispersed form, which was also reflected by their surface charge (Figure 4b).

The size and distribution range of the ZnO-NPs in the presence of 1, 5 and 10 mg/L HA decreased after 1 week of interaction compared to after 1 day (Figure 4a and Figure S5). However, after 2 and 3 weeks of interaction, the samples might be very polydispersed, and the particle size was not suitable for measurement by a dynamic light scattering analyzer (a scattered fraction of the samples was observed). Similar behaviour of the ZnO-NPs was observed in the presence of various concentrations of PFOA (i.e., 10 and 500 µg/L) with HA (Figure 4a and Figure S5). It is quite possible that HA (a large aliphatic network of carbon molecules) capped the effective edges of the nanoparticles, which ultimately caused their dispersion.

The presence of both PFOA and HA altered the size of the ZnO-NPs differently than the presence of individual PFOA or HA. Figure 4a and Figure S5 illustrate the size of the ZnO-NPs at the peak of the particle size distribution curve (PSDC (d, nm)) in the presence of various concentrations of HA and PFOA after various durations of interaction. The particle size and range of the ZnO-NPs increased (agglomerated particles) alone and in the presence of PFOA and decreased (polydispersed) at various concentrations (1, 5, and 10 mg/L) of HA. This dispersion behaviour of the ZnO-NPs may be associated with the presence of organic acids (i.e., HA), which may impact engineered ZnO-NPs by reducing their aggregation behaviour [19,60].

The number of specific affinity sites and the affinity coefficient of specific sites for organic pollutants are deemed to be the main influential parameters on the adsorbent capacity to deal with pollutants. Enhanced nanoparticle (ZnO) stability in suspension media by adsorbed dissolved organic matter can increase the total number of specific affinity sites, which supports the adsorption of organic pollutants (PFOA) on the surface of dissolved organic materials rather than on the nanoparticle surface. Simultaneously, the adsorbed organic matter may also produce new affinity sites and/or block the nanoparticle affinity sites to alter their capacity to adsorb pollutants. Dissolved organic matter, which is not adsorbed by nanoparticles, may also first adsorb pollutants and, second, curtail further adsorption of pollutants on the nanoparticle surface [15,38]. 

Zeta potential: The zeta potentials of the ZnO-NPs in buffered water, with PFOA, and with 1, 5, and 10 mg/L HA were measured after 1 day, 1, 2, and 3 weeks of interaction, respectively (Figure 4b). The magnitude of the surface charge of the ZnO-NPs alone and in the presence of PFOA decreased (from 1 day to 3 weeks) from −47.7 to −3.0 mV for the ZnO-NPs, from −48.6 to −3.1 mV for the ZnO-NPs with 10 µg/L PFOA, and from −47.9 to −3.5 mV for the ZnO-NPs with 500 µg/L PFOA. This behaviour confirmed the increase in the size of the nanoparticles due to agglomeration/sedimentation, which resulted in a reduced net charge (less negative) on the surface of the nanoparticles. However, the presence of HA decreased the magnitude of the change in the surface charge of the ZnO-NPs compared to that of the non-HA-containing samples. However, a high concentration of HA remained dominant in restraining the decrease in the magnitude of the zeta potential of the samples compared to that of lower HA concentrations (such as 1 mg/L HA) (Figure 4b) [18].

The aforementioned electrical potential data revealed that the aggregation behaviour of the pure ZnO-NPs in aqueous systems could be due to electrostatic interactions, van der Waals forces, and hydrophobic interactions. The environmental aging of nanoparticles alone and in the presence of organic pollutants, such as PFOA, could decrease the surface charge of the nanoparticles, increasing their sedimentation in environmental waters by decreasing their stability in aqueous systems. The presence of HA altered the surface charge in the reverse pattern compared to that of pure ZnO-NPs with and without the presence of PFOA. The HA substances covered the surface/effective sites of the nanoparticles because of their high aliphatic carbon content, which resulted in the least possibility of PFOA adsorbing on the nanoparticle surfaces. This also leads to the dispersion of the nanoparticles.

Adsorption: The PFOA in solution was measured to examine the sorption of PFOA with the ZnO-NPs alone and in the presence of various concentrations of HA after two weeks of interaction (Figure S6). An increase in the amount of adsorbed PFOA (10 and 500 µg/L) was calculated for ZnO NPs without HA after 2 weeks of interaction. However, the adsorption of PFOA decreased with the addition of high HA concentrations (such as from 1 to 5 and 10 mg/L HA). Dissolved humic acids can foul the adsorption of organic chemicals to microporous activated carbon through direct competition for adsorption sites and pore blockage [61].

Perfluoroalkyl acids, such as PFOA, contain a negatively charged hydrophilic head group and a hydrophobic–oleophobic perfluoroalkyl chain. Accordingly, a variety of mechanisms might be involved in the adsorption of PFOA in response to the surface properties (such as the charge and hydrophobicity) of adsorbents. The surface of HA is dominated by graphitic carbons, which are highly hydrophobic and have large electronic polarizability. Adsorption of PFOA molecules to HA is expected to be driven mainly by hydrophobic effects, which are combinations of entropic gradients and van der Waals (mainly dispersion) interactions between the adsorbate and adsorbent, whereas electrostatic forces play only a minimal role here. The low adsorption affinity of high concentrations of HA for PFOA is likely due to the low electronic polarizability of these molecules, thus decreasing potential van der Waals interactions despite the large electronic polarizability of graphitic carbons [62].

It could also be assumed that the highly aliphatic structure of HA dispersed the ZnO NPs, providing fewer active sites for the attachment of PFOA molecules. This also supported the results obtained (such as decreased zeta potential values for ZnO NPs alone and in the presence of 10 and 500 µg/L PFOA compared to samples with HA) in Figure 4a,b and Figure S6.

Dissolution: The dissolution of ZnO-NPs alone or in the presence of various concentrations of HA was observed in Milli-Q water at pH 7 controlled by using buffer solution (Figure S7). The presence of zinc in its dissolved or ionic form is potentially toxic to microorganisms, such as microflora [63,64]. The dissolution of ZnO-NPs can be influenced by the presence of other compounds in water [65], such as HA. The dissolved zinc concentration (mg/L) from ZnO-NPs alone or from ZnO-NPs combined with PFOA was calculated with the addition of various concentrations of HA in this study (Figure S7). After 1 day of interaction, the dissolved zinc concentration was greater in the presence of various concentrations of HA than in the absence of HA, and this trend was observed even after 1 week of interaction. It could be assumed that HA dispersed the nanoparticles after a long interaction time (such as 2–3 weeks), decreasing this dispersion effect by dominating the electrostatic forces, van der Waals forces, and hydrophobic interactions. This could also be caused by complexation (for zinc ions) with anionic HA followed by its large complex structure. Therefore, our findings are the same as those hypothesized by [63], i.e., that HA binds zinc ions.

TEM and XRD analysis: The samples from ZnO-NPs alone or in the presence of PFOA or HA were analyzed using TEM morphology and elemental mapping after immediate preparation (such as after 0 h of interaction) and after 1 day of interaction (Figure 4c). ZnO-NPs were more aggregated after 1 day of interaction than after 0 h, which is consistent with previous findings (Figure 1a). TEM revealed an increase in the size of the ZnO-NPs in the presence of 10 mg/L PFOA (only this concentration was selected for TEM analysis to confirm the presence PFOA, such as fluorine in mapping) after 1 day of interaction compared to 0 h. Elemental mapping further confirmed the presence of Zn, O, F, P, and K (Figure S8). The dispersion patterns of ZnO-NPs, alone and in the presence of PFOA, due to the presence of highly aliphatic and complex structures of HA, can be observed (Figure 4c) when comparing images from 0 h and 1 day. The SAED images did indicate less crystallinity on the nanoparticles after 1 day of interaction, which matches the results obtained from the zeta analysis. Elemental mapping further confirmed the presence of the expected elements. Figure S8 shows the elemental composition comparisons of the ZnO-NPs with contaminants at different intervals.

Post-photocatalysis characterization, namely XRD (Figure S8b), of ZnO-NPs, HA, and ZnO-NPs with and without the presence of 10 mg/L PFOA and 10 mg/L HA was performed to further examine the impact of PFOA and HA on the crystallinity and purity of ZnO-NPs. It was observed that there was no change in the crystal phase of the ZnO-NPs after 1 day of interaction. However, after 1 week of interaction (Figure S8b), the intensities of the peak alignments at (1 0 0), (0 0 2), and (1 0 1) were suppressed. Four new peaks at 2θ values of 9.68° and 19.36°, 22.58°, and 25.6° were observed, indicating signs of alterations into the crystallinity and purity of the ZnO-NPs influence by the adsorption of co-contaminants and aging factors.

### 3.4. Influence of Electrolytes and HA Together on PFOA and ZnO-NPs’ Interaction

**Figure 5:**
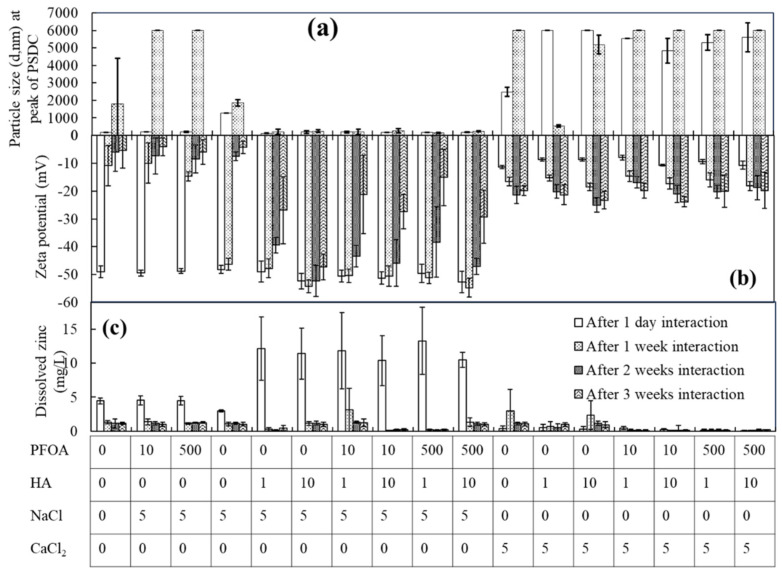
The particle size (**a**), zeta potential (**b**), and dissolved Zn (**c**) analysis for thr interaction of ZnO-NPs with PFOA under various conditions; concentrations for PFOA, HA, NaCl, and CaCl_2_ are µg/L, mg/L, mM, and mM respectively. Zeta potential values are minus”.

A mixture of salts and dissolved organic matter could influence the particle size and surface charge differently, which was investigated and explained in this section (Figure 5).

Particle size: The sizes of the ZnO-NPs at the peak of PSDC (d, nm) without and with the presence of PFOA and with 5 mM concentrations of monovalent and divalent electrolytes and various concentrations of HA after 1 day, 1 week, 2 weeks, and 3 weeks of interaction are illustrated (Figure 5 and Figure S9). With the increasing size of ZnO-NPs alone or with PFOA and the addition of 5 mM concentrations of monovalent salt (NaCl) and divalent CaCl_2_, the size of the ZnO-NPs increased as the interaction time increased, such as from 1 day to 1 week. The size was out of the machine range (range:$\;0.3–1.0\times 10^{4}\;nm$) after 1 week due to the aggregation of nanoparticles, and only a few fragments were measured after 1 week of interaction. It is assumed that the particle size at the peak of the PSDC would be larger than 10,000 nm. In the presence of HA, initial dispersion was observed with increasing HA concentration in the presence of salts and PFOA; however, after a few weeks, the same behaviour of aggregation/agglomeration/sedimentation was observed, with the particle size being nonuniform and not measurable, while there was a decreasing zeta potential.

These findings revealed that the influence of humic substances on the interactions between PFOA and ZnO-NPs is somewhat complicated, especially when electrolytes are present. Counteractions for PFOA between HA and ZnO-NPs could reduce the effective interactions of ZnO-NPs by decreasing the amount of PFOA available for sorption. Moreover, natural organic matter can cover the surface of nanoparticles and thereby reduce their affinity for organic pollutants [38,57,66]. 

Zeta potential: A decrease in the magnitude of the surface charge of ZnO NPs alone or in the presence of PFOA or salt was observed after 1 day to 3 weeks of interaction, as shown in Figure 5b. This indicated that the ZnO-NPs exhibited a similar agglomeration (size increase) behaviour (Figure 5) with a diminished surface charge, as described in the aforementioned sections. The addition of various concentrations of HA in the presence of PFOA did not significantly change the zeta potential compared to that of samples with HA. This confirms that interactions between HA molecules (the dispersion of HA-coated ZnO-NPs) are more dominant at high concentrations (such as 10 mg/L HA) than the electrostatic interactions between negatively charged nanoparticles surrounded by monovalent cations (Na^+^). Conversely, divalent cations interacted more strongly with negative surface charges, and the overall zeta potential decreased in magnitude in the presence of both divalent cations (Ca^2+^) (Figure 5b). However, the dispersion effect of HA on ZnO-NPs was also observed when PFOA and divalent electrolyte (CaCl_2_) salts were present (Figure 5b), as noted in the findings. It could be inferred that salinity and natural organic substances play significant roles in the transport of ZnO-NPs and their associated organic pollutants (PFOA) from fresh water to the ocean, especially in estuary regions.

Adsorption: Adsorption of PFOA was examined for ZnO-NPs alone or in the presence of various concentrations of HA in the presence of 5 mM NaCl and CaCl_2_ after two weeks of interaction (Table S1). An increase in the amount of adsorbed PFOA (10 and 500 µg/L) was calculated for ZnO NPs without HA after 2 weeks of interaction. However, the adsorption of PFOA decreased with the addition of high HA concentrations (such as 1 and 10 mg/L HA) (Table S1). It could be assumed that the highly aliphatic structure of HA dispersed the ZnO NPs, providing fewer active sites for the attachment of PFOA molecules. This result also supported the results obtained, i.e., decreased zeta potential values for ZnO NPs alone and in the presence of 10 and 500 µg/L PFOA compared to samples with HA in Figure 5b.

Dissolution: The dissolved zinc concentration (mg/L) from ZnO-NPs alone, with PFOA and salts, and with the addition of various concentrations of HA, was measured in this study (Figure 5c). After 1 day of interaction, the dissolved zinc concentration was greater in the presence of various concentrations of HA than in the absence of HA. It could be assumed that HA dispersed the nanoparticles; however, after a long interaction time (such as 1, 2, or 3 weeks), the dispersion effect decreased due to the dominant electrostatic forces, van der Waals forces, and hydrophobic interactions.

TEM analysis: The morphological behaviour of ZnO-NPs with PFOA, HA, and CaCl_2_ after 0 h and 1 day intervals was examined via TEM (Figure S10). The particles were aggregated after 0 h in the presence of 5 mM CaCl_2_. However, after 1 day of interaction, shaded (due to CaCl_2_) and dispersed (due to HA) patterns of the nanoparticles can be observed. The bright diffraction signals are due to the crystalline ZnO-NPs, including the presence of CaCl_2_ crystals. To examine the morphological changes associated with high concentrations of CaCl_2_, 10 mM CaCl_2_ (after 0 h of interaction) was added, which generated clusters/agglomerates. However, after 1 day of interaction, the nanoparticles were dispersed by coating them with large HA molecules (Figure S10d).

**Figure 6:**
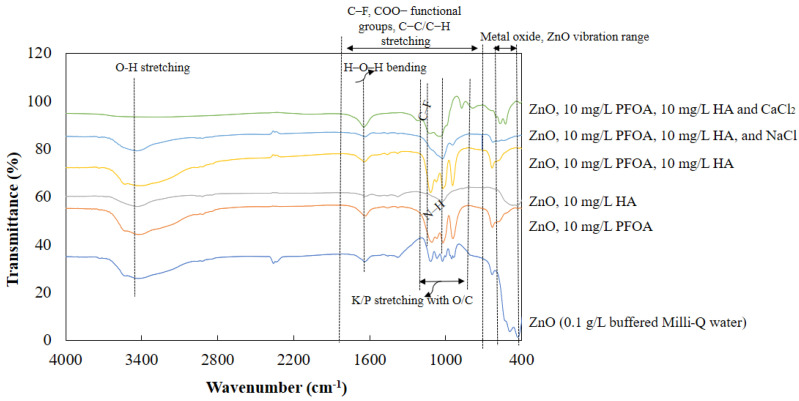
FTIR analysis of interactions between ZnO-NPs and PFOA in the presence of HA and electrolytes.

FTIR: The interactions of ZnO-NPs with PFOA in the presence of HA and electrolytes were investigated via FTIR analysis (Figure 6). The metal oxide (ZnO) absorbance ranged from 600 to 400 cm^−1^ [15,49,67], which indicated the presence of interacting ZnO-NPs. This difference was detected in all the samples, while the intensity of the absorbance peak depth and location varied. Peaks at 1800 and 600 cm^−1^ represent carboxylate functional groups and C−F [51], C−C, and C−H stretching, respectively [50]. In particular, the vibrational peak at approximately 1102 cm^−1^ appeared in all samples with PFOA, representing the presence of C–F stretching bonds. This indicated the interaction between PFOA and the ZnO-NPs. The absorbance at 1645 cm^−1^ is due to H−O−H bending. The infrared band at 1010 cm^−1^ in all the examples except for ZnO-NPs and ZnO + PFOA is due to the stretching of N–H bonds [50] present in an organic compound, such as HA. The peaks between 1050 and 750 cm^−1^ could be due to K-potassium and P-phosphorous (from a buffer solution used to maintain pH 7) stretching with O and C. The absorbance at 3490 cm^−1^ is due to O-H stretching [50]. The peaks at approximately 933 and 871 cm^−1^ could be due to triatomic inorganic molecules (calcium chlorine), while 670 cm^−1^ could be due to CO_2_ from the atmosphere [50]. 

### 3.5. Interaction Scheme

**Figure 7:**
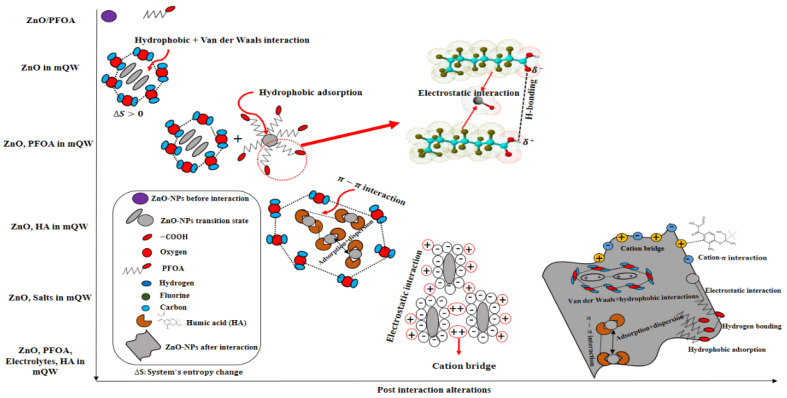
Possible potential interaction mechanisms between ZnO-NPs (size ≤ 100 nm) and PFOA in the presence of key factors (e.g., natural organic substances and electrolytes). The + and – symbols represent positive and negative charges respectively.

A schematic diagram illustrating the interaction mechanisms is shown in Figure 7, depicting the proposed interactions between ZnO-NPs and PFOA and HA in the absence and presence of cations in simulated waters under controlled laboratory conditions. ZnO-NPs tend to aggregate in aqueous media (0.1 g/L) due to van der Waals and hydrophobic interactions surrounded by hydrogen bonding between water molecules (Figure 1 and Figure 7). The hydrophobic molecules of PFOA could be comparatively easily adsorbed on the porous surface of the ZnO-NPs, which subsequently enhanced the size of the nanoparticles through electrostatic interactions, hydrogen bonding and van der Waals interactions. Specific surface area and surface morphology of the ZnO-NPs are key factors that impact the interaction mechanisms between ZnO-NPs and PFOA. The roughness, specific surface area, porosity, and shape and size of ZnO-NPs may significantly affect the interactions of PFOA molecules with ZnO-NPs. For instance, porous surfaces with more irregularly sized crystal structures, may provide more active sites for the sorption of PFOA molecules. The surface energy of ZnO-NPs is also associated with their surface morphology. The higher the surface energy, the stronger the interactions with PFOA molecules, leading to more adsorption. However, both the specific surface area and surface morphology of the ZnO-NPs influence the interaction mechanisms (such as adsorption) of PFOA onto ZnO NPs. Van der Waals and hydrophobic interactions may cause the nanoparticles to agglomerate. The coexistence of electrolytes in water systems can screen the charge on the surface of nanoparticles by counter ions and consequently make them unstable at high salt concentrations, in addition to agglomerating. The electrical potential of the ZnO-NPs was changed by varying the type of electrolyte used during the reaction. The type of electrolyte affects the alterations in the surface charge of the ZnO-NPs differently. The aggregation of ZnO-NPs in the presence of monovalent and divalent electrolytes is influenced by the surface charge via electrostatic interactions. Furthermore, cation bridging and ligand binding could also be considered.

The addition of HA dispersed the ZnO-NPs by coating them (due to their large molecules), which meant that the ZnO-NPs had the least opportunity to attach to other contaminants. The presence of salts promoted electrostatic interactions and bridging effects as well. Moreover, the presence of PFOA, HA, and salts influenced the surface charge by adsorbing on the active surfaces, covering the surface made possible by the complex and large structure of HA molecules and dispersing nanoparticles and cation bridging, respectively. These factors could lead to alterations in the particle size and morphology of the ZnO-NPs. In summary, once ZnO-NPs are released in ecosystems containing various types of organic and inorganic pollutants, humic substances, and electrolytes, alterations in the parental nanoparticles can be expected. This is in terms of their agglomeration state, crystallinity morphology, purity, size, and surface chemistry. Comparison between this study with previous studies for the responsible interaction mechanisms are tabulated (Table S2).

## 4. Conclusions

This study revealed that the size, shape, crystallinity, surface charge, and morphology of the ZnO-NPs were altered after they interacted with PFOA, coexisting HA, and salts for various durations as aging factors under controlled pH (i.e., pH 7) in simulated water systems. Due to its large complex structure, humic acid behaves as a dispersant by covering ZnO-NPs and leaving the least opportunity for other compounds to adsorb. Variations in the size, shape, and surface charge due to aging of the ZnO-NPs could also be key factors in their fate and behaviour in combination with other environmental factors. PFOA-sorbed ZnO-NPs may slowly sink and reach sediments in the form of agglomerates in the presence of other substances in water media, such as altered/toxic substances. In addition to this, a toxicity study is recommended in future to determine the toxic impacts of PFOA-adsorbed ZnO-NPs on the living organisms.

## Data Availability

All relevant data are reported in main text or Supplementary Information.

## Supplementary Materials

The following supporting information can be downloaded at: https://www.mdpi.com/article/10.3390/toxics12080602/s1, Figure S1: Particle size distribution of ZnO-NPs after 1 day (a) and 1 week (b) of interaction with PFOA; ZnO-NPs 0.1 g/L, pH 7; Figure S2a: TEM elemental analysis of ZnO-NPs with PFOA after 1 day of interaction; Figure S2b: TEM analysis (lattice spacing, crystallite size) of ZnO-NPs with PFOA after 1 day of interaction; Figure S3: Concentration of dissolved zinc at pH 7 after 1 day and 2 weeks of interaction; Figure S4: Particle size distribution of ZnO-NPs after 1 day (a) and 1 week (b) of interaction with PFOA in the presence of electrolytes at pH 7 and room temperature (i.e., 20 ℃); Figure S5: Particle size distribution of ZnO-NPs after 1 day (a) and 1 week (b) of interaction in the presence of various concentrations of PFOA and HA at pH 7 and room temperature (i.e., 20 ℃); Figure S6: Adsorption of PFOA on the surface of ZnO-NPs after 2 weeks of interaction in the presence of various concentrations of PFOA and HA; Figure S7: Dissolved zinc (mg/L) at pH 7 after 1 day, 1 week, 2 weeks, and 3 weeks of interaction with various concentrations of PFOA and HA; Figure S8a: Elemental composition comparisons of the ZnO-NPs with contaminants; Figure S8b: XRD analysis of ZnO-NPs powder, HA powder, ZnO-NPs with and without interactions of 10 mg/L PFOA and 10 mg/L HA after 1 day and 1 weeks of interactions; Figure S9: Particle size distribution of ZnO-NPs after 1 day (a) and 1 week (b) of interaction with PFOA in the presence of electrolytes and HA at pH 7 and room temperature (i.e., 20 ℃); Figure S10: TEM images of ZnO NP under various concentrations of CaCl_2_ after 0 h and 24 h of interaction in solution (drops taken on a TEM grid from solution); ZnO, 10 mg/L PFOA, 10 mg/L HA, 5 mM CaCl_2_ (a,b); ZnO, 10 mg/L PFOA, 10 mg/L HA, and 10 mM CaCl_2_ (c,d); Table S1: Adsorption of PFOA on ZnO NPs (mg/g) in the presence of electrolytes and various concentrations of HA; Table S2: Interaction mechanisms of engineered nanoparticles with organic substances. References [15,22,24,25,68] are cited in the supplementary materials.
