# Supplementary material for: Changes in the Aggregation Behaviour of Zinc Oxide Nanoparticles Influenced by Perfluorooctanoic Acid, Salts, and Humic Acid in Simulated Waters
Source: Toxics. 2024 Aug 18;12(8):602. doi: 10.3390/toxics12080602 (PMC11359926; doi:10.3390/toxics12080602)
Supplement: Supplementary file 1 [file toxics-12-00602-s001.zip › toxics-3108764-supplementary.pdf]

Supplementary information

List of figures and tables

Figure S1. Particle size distribution of ZnO-NPs after 1 day (a) and 1 week (b) of interaction with PFOA; ZnO-NPs 0.1 g/L, pH 7. 2
Figure S2a.TEM elemental analysis of ZnO-NPs with PFOA after 1 day of interaction. 3
Figure S2b.TEM analysis (lattice spacing, crystallite size) of ZnO-NPs with PFOA after 1 day of interaction. 3
Figure S3. Concentration of dissolved zinc at pH 7 after 1 day and 2 weeks of interaction. 4
Figure S4. Particle size distribution of ZnO-NPs after 1 day (a) and 1 week (b) of interaction with PFOA in the presence of electrolytes at pH 7 and room temperature (i.e., 20 ℃). 5
Figure S5 Particle size distribution of ZnO-NPs after 1 day (a) and 1 week (b) of interaction in the presence of various concentrations of PFOA and HA at pH 7 and room temperature (i.e., 20 ℃). 6
Figure S6. Adsorption of PFOA on the surface of ZnO-NPs after 2 weeks of interaction in the presence of various concentrations of PFOA and HA. 7
Figure S7. Dissolved zinc (mg/L) at pH 7 after 1 day, 1 week, 2 weeks, and 3 weeks of interaction with various concentrations of PFOA and HA. 8
Figure S8a. Elemental composition comparisons of the ZnO-NPs with contaminants. 9
Figure S9. Particle size distribution of ZnO-NPs after 1 day (a) and 1 week (b) of interaction with PFOA in the presence of electrolytes and HA at pH 7 and room temperature (i.e., 20 ℃). 10
Figure S10. TEM images of ZnO NP under various concentrations of $CaCl_2$ after 0 h and 24 h of interaction in solution (drops taken on a TEM grid from solution); ZnO, 10 mg/L PFOA, 10 mg/L HA, 5 mM $CaCl_2$ (a-b); ZnO, 10 mg/L PFOA, 10 mg/L HA, and 10 mM $CaCl_2$ (c-d). 11

Table S1. Adsorption of PFOA on ZnO NPs (mg/g) in the presence of electrolytes and various concentrations of HA 12
Table S 2 Interaction mechanisms of engineered nanoparticles with organic substances 13

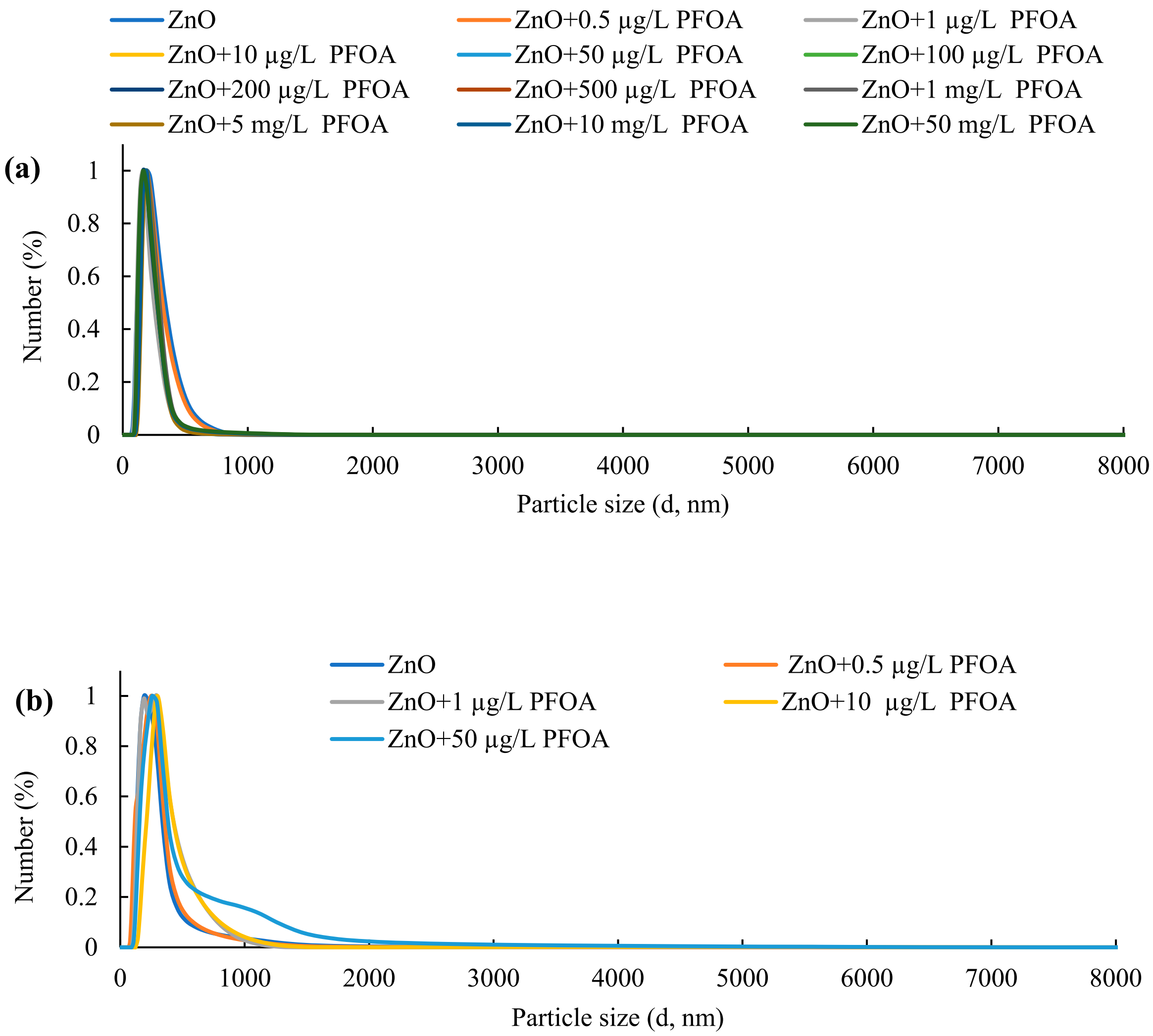

Figure S1. Particle size distribution of ZnO-NPs after 1 day (a) and 1 week (b) of interaction with PFOA; ZnO-NPs 0.1 g/L, pH 7.

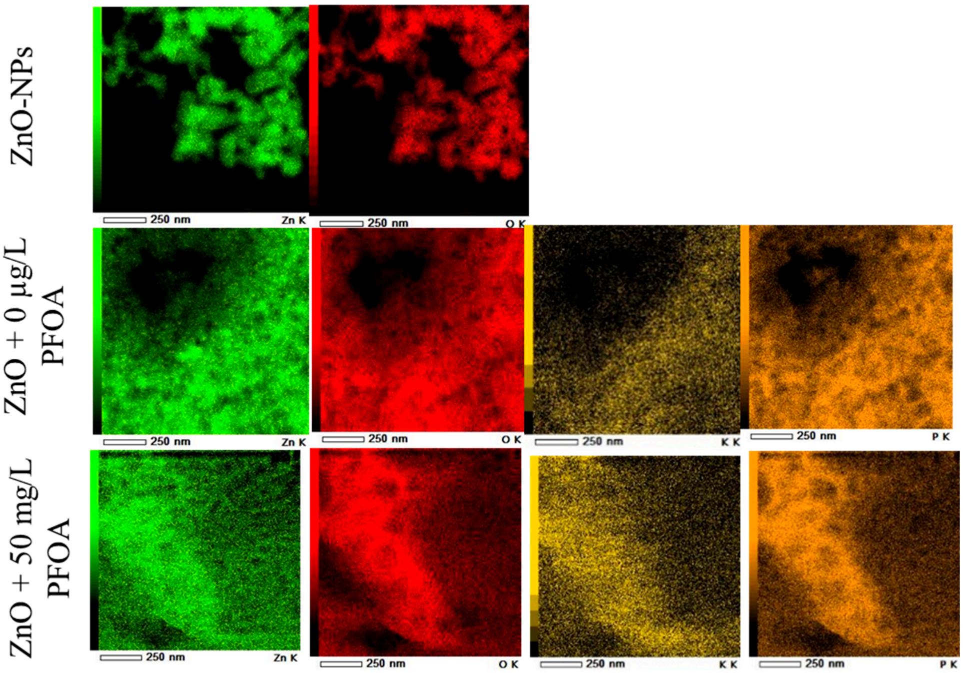

Figure S2a.TEM elemental analysis of ZnO-NPs with PFOA after 1 day of interaction.

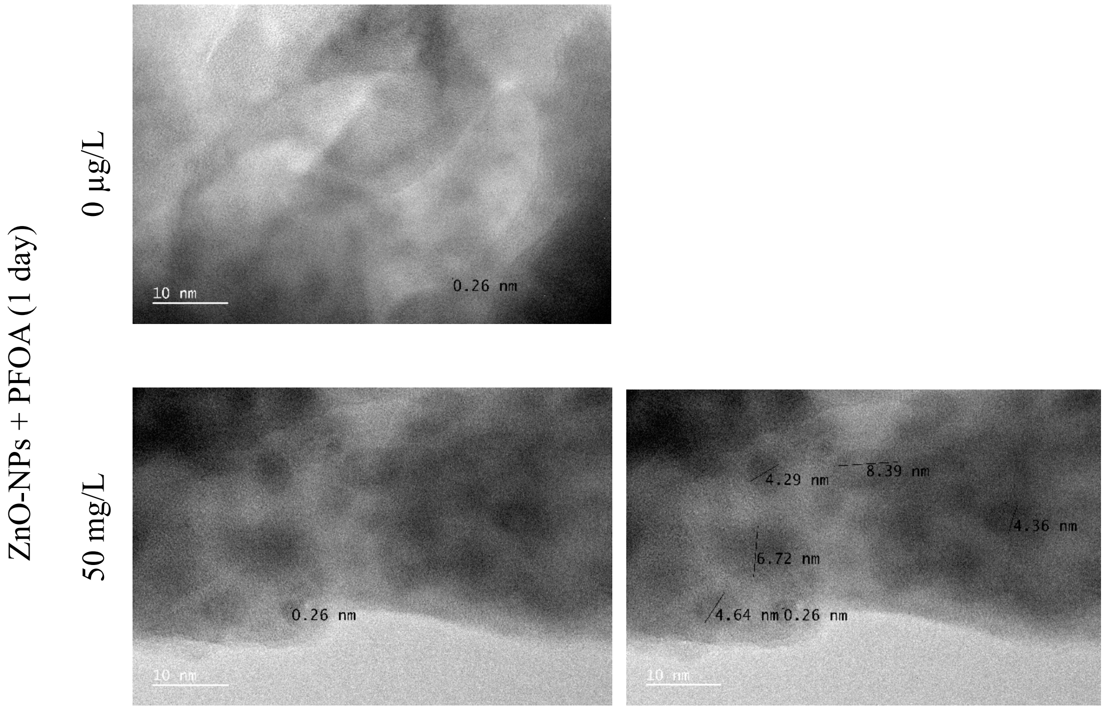

Figure S3b.TEM analysis (lattice spacing, crystallite size) of ZnO-NPs with PFOA after 1 day of interaction.

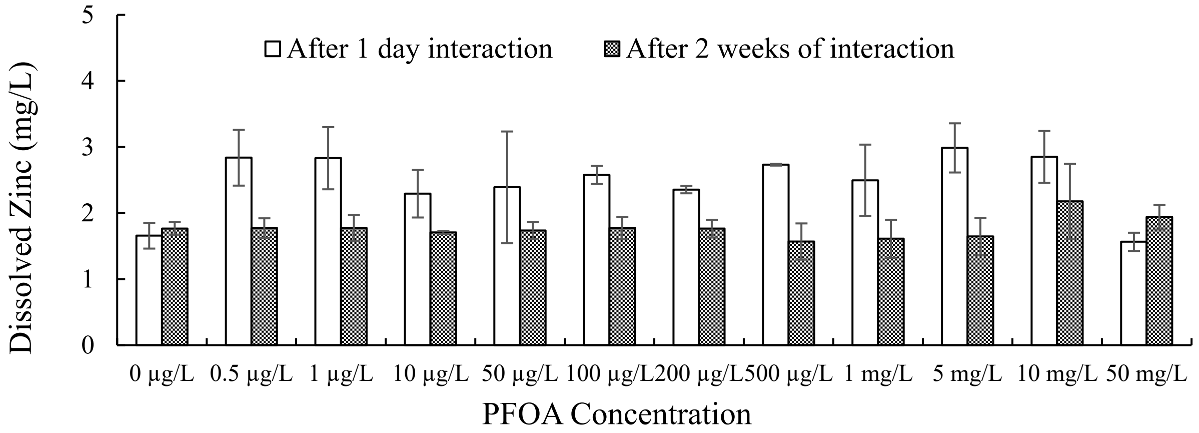

Figure S4. Concentration of dissolved zinc at pH 7 after 1 day and 2 weeks of interaction.

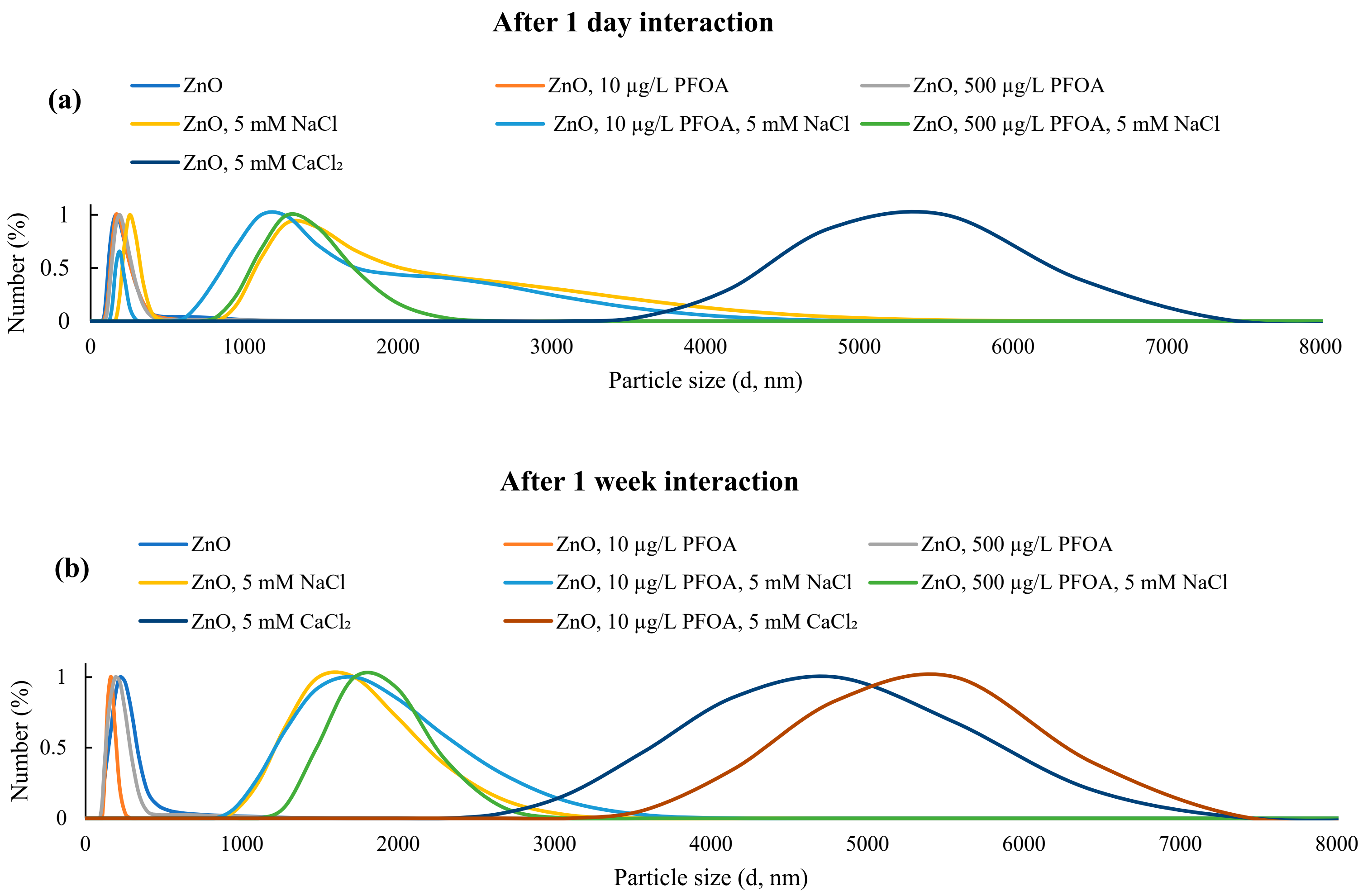

Figure S5. Particle size distribution of ZnO-NPs after 1 day (a) and 1 week (b) of interaction with PFOA in the presence of electrolytes at pH 7 and room temperature (i.e., 20 °C).

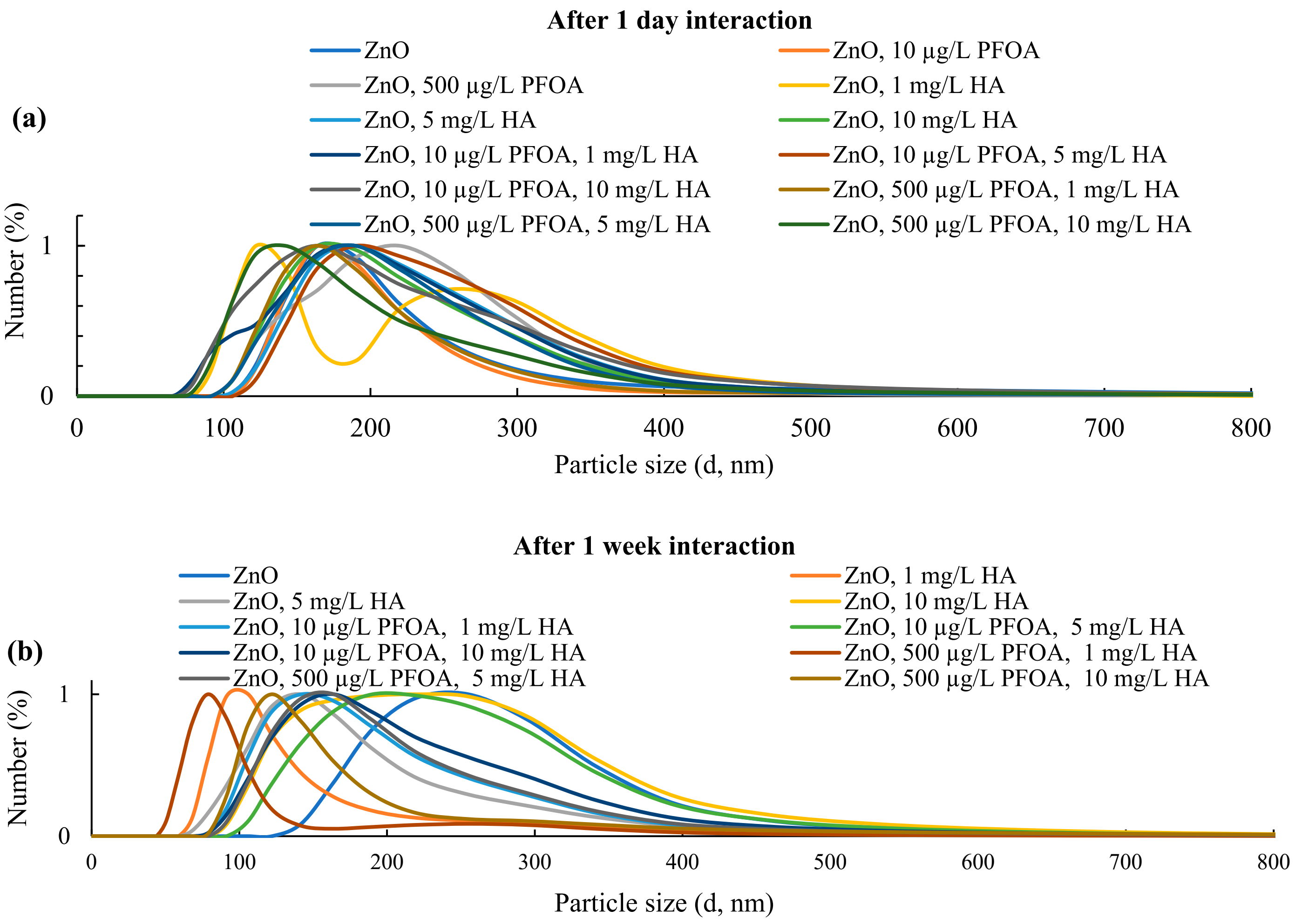

Figure S6 Particle size distribution of ZnO-NPs after 1 day (a) and 1 week (b) of interaction in the presence of various concentrations of PFOA and HA at pH 7 and room temperature (i.e., 20 °C).

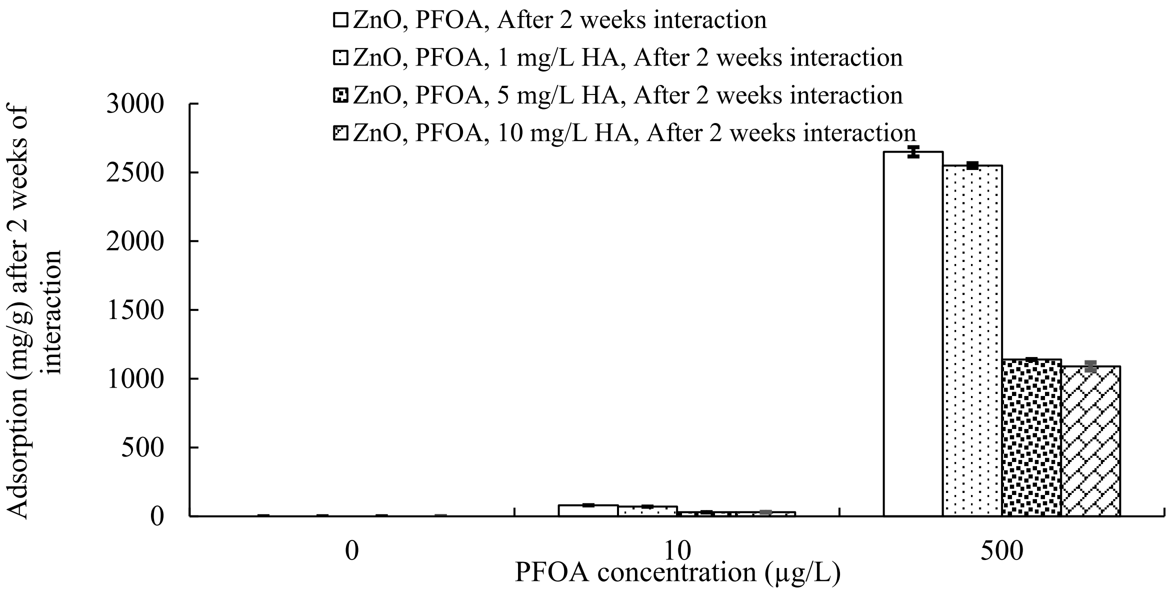

Figure S7. Adsorption of PFOA on the surface of ZnO-NPs after 2 weeks of interaction in the presence of various concentrations of PFOA and HA.

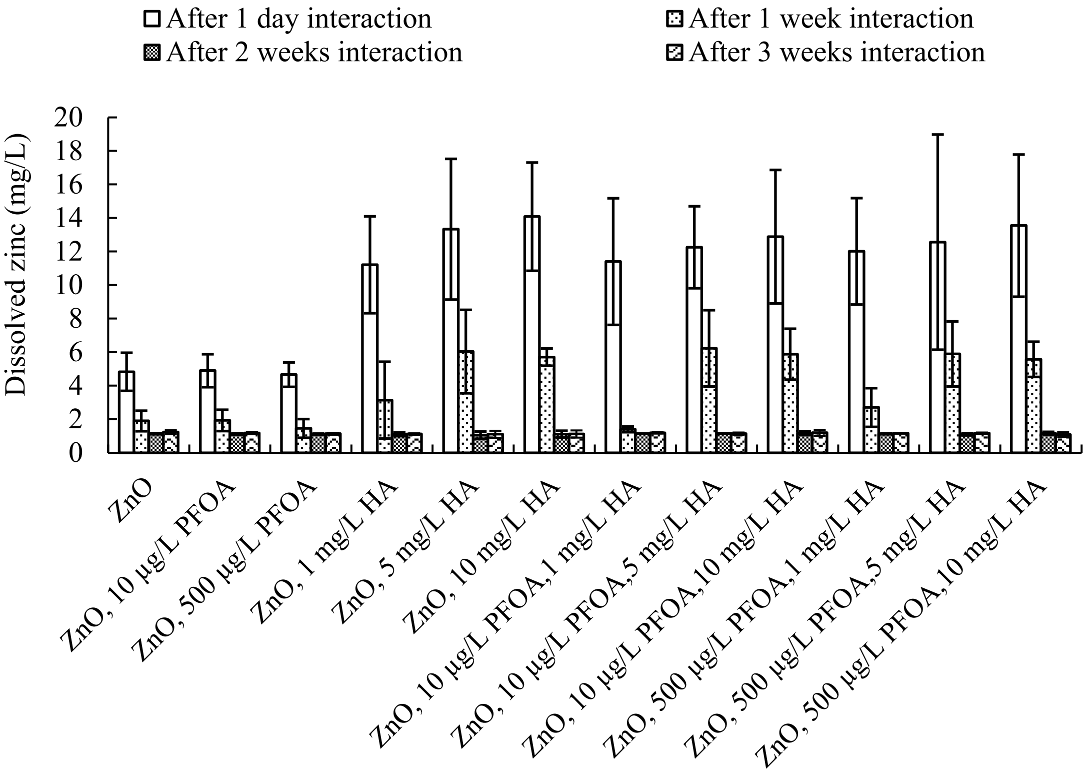

Figure S8. Dissolved zinc (mg/L) at pH 7 after 1 day, 1 week, 2 weeks, and 3 weeks of interaction with various concentrations of PFOA and HA.

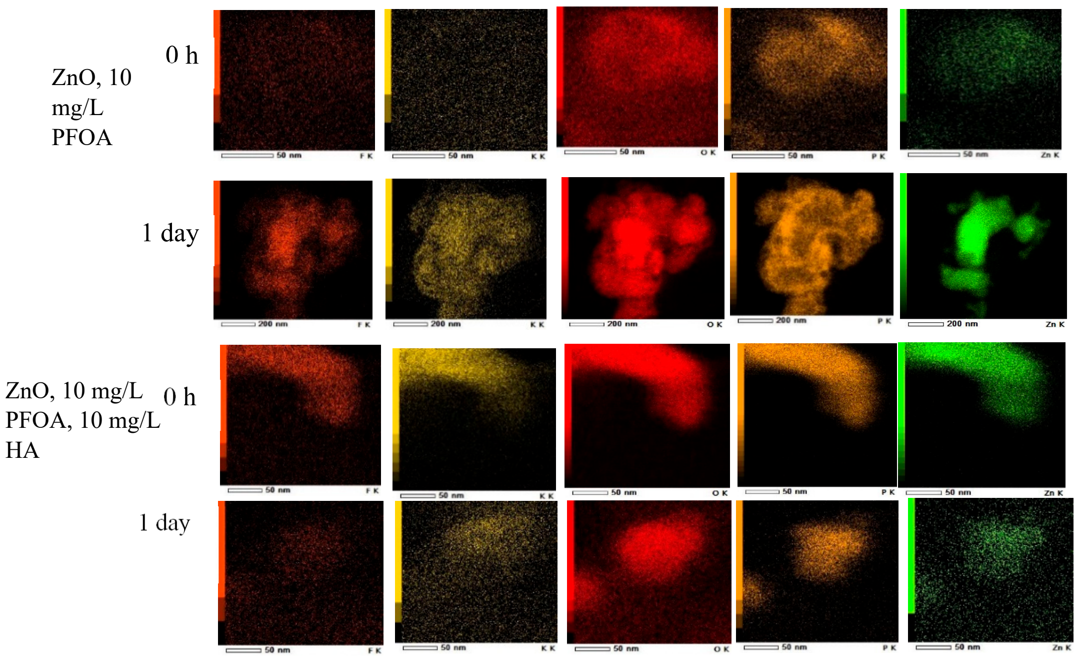

Figure S9a. Elemental composition comparisons of the ZnO-NPs with contaminants.

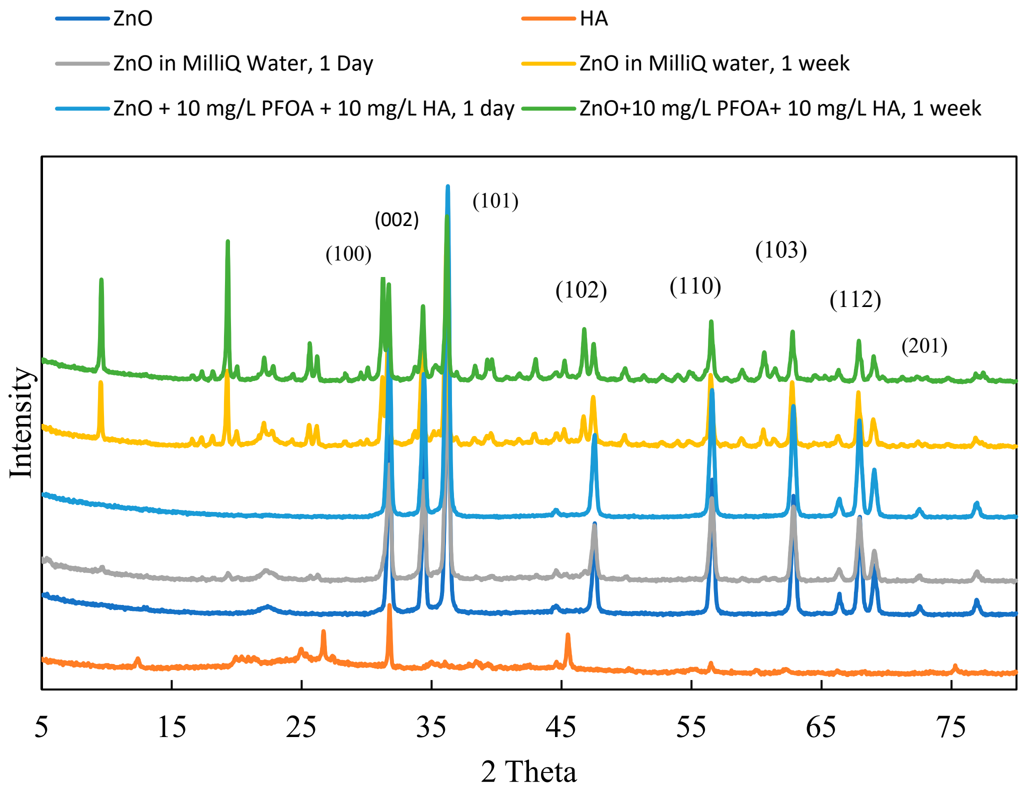

Figure S8b. XRD analysis of ZnO-NPs powder, HA powder, ZnO-NPs with and without interactions of 10 mg/L PFOA and 10 mg/L HA after 1 day and 1 weeks of interactions.

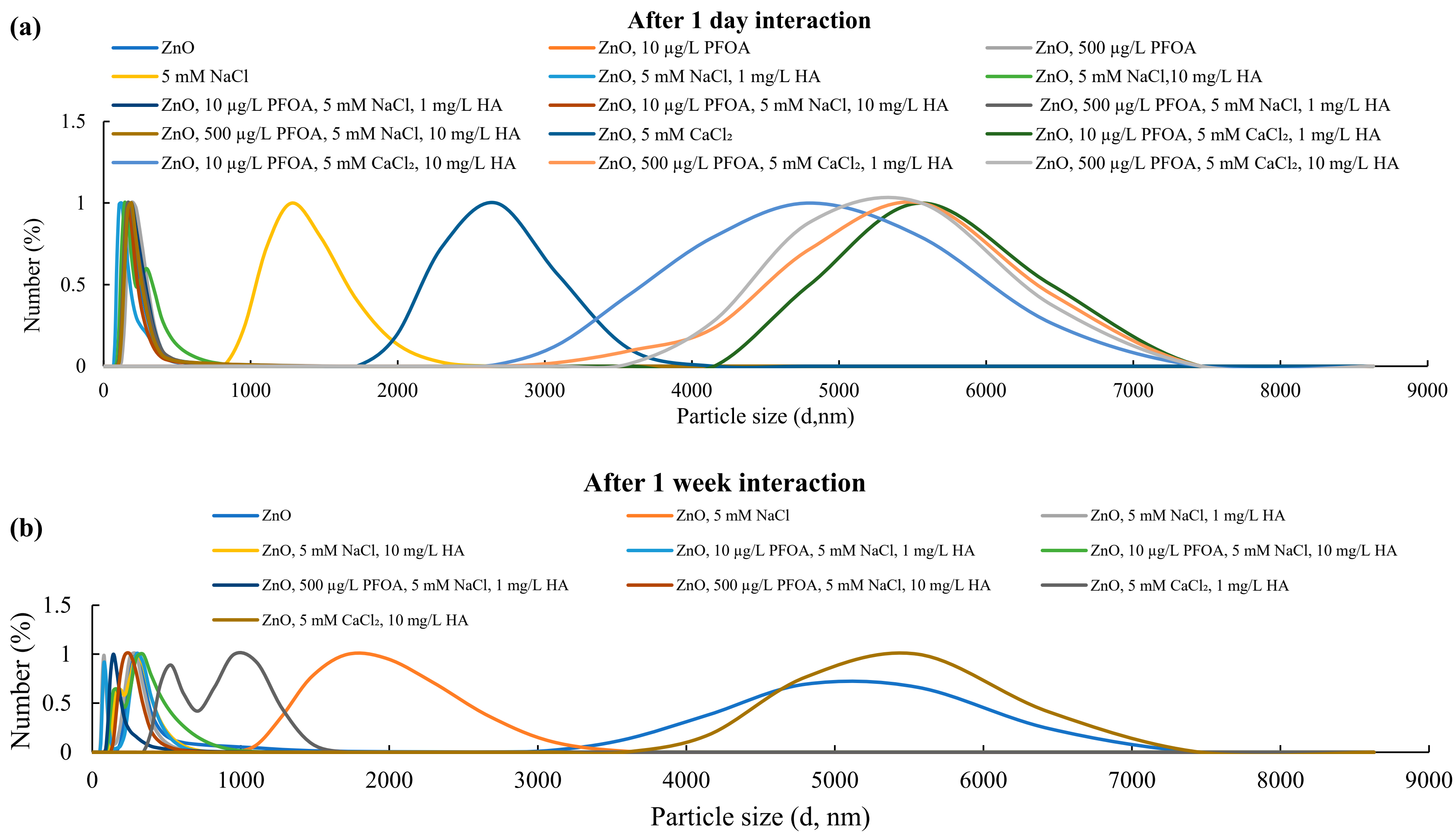

Figure S10. Particle size distribution of ZnO-NPs after 1 day (a) and 1 week (b) of interaction with PFOA in the presence of electrolytes and HA at pH 7 and room temperature (i.e., 20 °C).

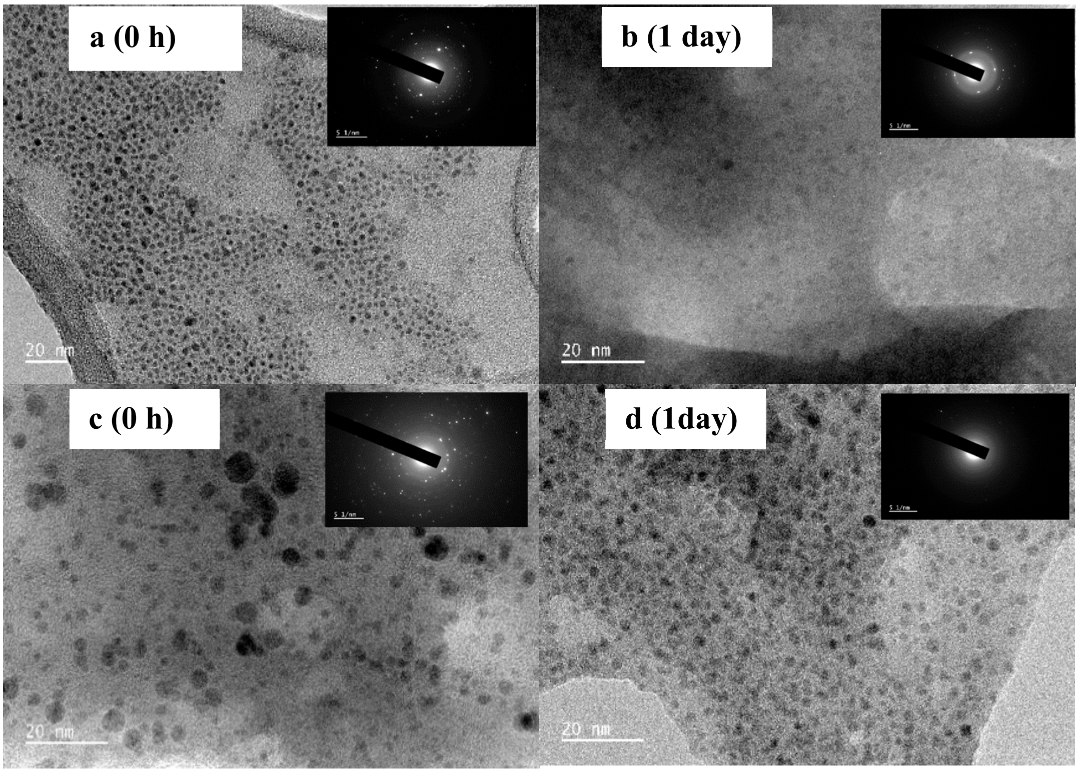

Figure S11. TEM images of ZnO NP under various concentrations of $CaCl_2$ after 0 h and 24 h of interaction in solution (drops taken on a TEM grid from solution); ZnO, 10 mg/L PFOA, 10 mg/L HA, 5 mM $CaCl_2$ (a-b); ZnO, 10 mg/L PFOA, 10 mg/L HA, and 10 mM $CaCl_2$ (c-d).

Table S1. Adsorption of PFOA on ZnO NPs (mg/g) in the presence of electrolytes and various concentrations of HA

**PFOA (μg/L)**	0	10	500	10	10	500	500	10	10	500	500
**NaCl (mM)**	0	0	0	5	5	5	5	0	0	0	0
**$CaCl_2$ (mM)**	0	0	0	0	0	0	0	5	5	5	5
**HA (mg/L)**	0	0	0	1	10	1	10	1	10	1	10
**PFOA sorbed (mg/g)**	0	96	3000	80	40	2490	1710	90	80	3570	2510

Table S 2 Interaction mechanisms of engineered nanoparticles with organic substances

Interacting Species	Responsible Interaction Mechanisms	Comments	References
Titanium oxide nanoparticles (TiO_2-NPs)	Hydrogen bonding, electrostatic interactions, van der Waals foces, hydrophobic and steric interactions	• Higher ionic concentration of electrolytes dropped the magnitude of zeta potential<br>• (surface charge) of titanium oxide nanoparticles with or without presence of polybroinated diphenyl ethers (such as BDE-47 and BDE-209).	[25]
Graphene	Electrostatic force of interactions, Van der Waals forces, steric hindrance, hydrophobic interactions, π-π stacking.	• The interaction mechanisms between polybrominated diphenyl ethers (PBDEs) onto the graphene surface were studies by applying Density functional theory (DFT) and molecular dynamics (MD) methods.<br>• Aromatic ring favours adsorption on graphene with a corresponding alignment to the graphene surface.<br>• Steric hinderance caused by the ortho-position substitution of bromine in BDE-183 and -209.	[24]
Dissolved organic substances	Mostly π-π interactions are affective.	• Substituent positions impact on the binding affinities of persistent organic pollutants.	[68]
Zinc oxide nanoparticles (ZnO-NPs) and hexabromocyclododecane (HBCD)	Hydrogen bonding, electrostatic and hydrophobic interactions.	• ZnO-NPs size increased (such as from nanometres to micrometres) with increased concentrations of HBCD. In parallel, magnitude of surface charge was also dropped with increased HBCD amount influence by aging factors as well. Humic acid dispersed the nanoparticles	[15]
Sunscreen extracted ZnO-NPs	Particle-particle interactions Van der Waals forces, hydrophobic interaction	• Particles size increased influence by aging factor	[22]
ZnO-NPs and perfluorooctanoic acid (PFOA)	Electrostatic force of interactions, Van der Waals forces, Particle-particle interactions, hydrophobic interaction, bridging interactions	• Particles agglomerated due to adsorption of PFOA molecules including particles-particles interactions as well. Aging influence on the adsorption of PFOA into the surface of ZnO-NPs.<br>• Electrolytes agglomerated the ZnO-NPs.<br>• Humic acid resistant the sedimentation making them to remain ZnO-NPs into suspension form compared to electrolytes.<br>• First time PFOA interaction was studied with ZnO-NPs explaining how the fate and behaviour of ZnO-	**This study**<br>**Advantage of our study**

	NPs affected by the adsorption of organic pollutant (PFOA), influence by electrolytes, and humic acid, and aging factors.	

The end of the supplementary information.
